# Decisive role of mDia-family formins in cell cortex function of highly adherent cells

**DOI:** 10.1126/sciadv.adp5929

**Published:** 2024-10-30

**Authors:** Jonas Scholz, Till Stephan, Aina Gallemí Pérez, Agnes Csiszár, Nils Hersch, Lisa S. Fischer, Stefan Brühmann, Sarah Körber, Christof Litschko, Lucija Mijanovic, Thomas Kaufmann, Felix Lange, Ronald Springer, Andreas Pich, Stefan Jakobs, Michelle Peckham, Marco Tarantola, Carsten Grashoff, Rudolf Merkel, Jan Faix

**Affiliations:** ^1^ Institute for Biophysical Chemistry, Hannover Medical School, Hannover, Germany.; ^2^ Department of NanoBiophotonics, Max Planck Institute for Multidisciplinary Sciences, Göttingen, Germany.; ^3^ Clinic of Neurology, University Medical Center Göttingen, Göttingen, Germany.; ^4^ Buchmann Institute for Molecular Life Sciences, Goethe University Frankfurt, Frankfurt/Main, Germany.; ^5^ Institute for Dynamics of Complex Systems, Göttingen, Germany.; ^6^ Max Planck Institute for Dynamics and Self-Organization, Department LFPB, Göttingen, Germany.; ^7^ Institute of Biological Information Processing 2: Mechanobiology, Forschungszentrum Jülich GmbH, Jülich, Germany.; ^8^ Institute of Integrative Cell Biology and Physiology, University of Münster, Münster, Germany.; ^9^ HiLIFE Institute of Biotechnology, University of Helsinki, Helsinki, Finland.; ^10^ Research Core Unit Proteomics and Institute of Toxicology, Hannover Medical School, Hannover, Germany.; ^11^ Astbury Centre for Structural Molecular Biology, University of Leeds, Leeds, UK.

## Abstract

Cortical formins, pivotal for the assembly of linear actin filaments beneath the membrane, exert only minor effects on unconfined cell migration of weakly and moderately adherent cells. However, their impact on migration and mechanostability of highly adherent cells remains poorly understood. Here, we demonstrate that loss of cortical actin filaments generated by the formins mDia1 and mDia3 drastically compromises cell migration and mechanics in highly adherent fibroblasts. Biophysical analysis of the mechanical properties of the mutant cells revealed a markedly softened cell cortex in the poorly adherent state. Unexpectedly, in the highly adherent state, associated with a hyperstretched morphology with exaggerated focal adhesions and prominent high-strain stress fibers, they exhibited even higher cortical tension compared to control. Notably, misguidance of intracellular forces, frequently accompanied by stress-fiber rupture, culminated in the formation of tension- and contractility-induced macroapertures, which was instantly followed by excessive lamellipodial protrusion at the periphery, providing critical insights into mechanotransduction of mechanically stressed and highly adherent cells.

## Teaser

mDia1/3 loss disrupts cortex mechanics, enhances matrix adhesion, and induces transcellular macroaperture formation

## INTRODUCTION

The contractile actin cortex is a thin, viscoelastic layer of cross-linked actin filaments, nonmuscle myosin 2, and associated proteins tethered to the plasma membrane of eukaryotic cells (*1*–*3*). Myosin-generated forces create tension in the cortical network, and gradients in cortical tension lead to cellular deformations such as those arising during mitosis, cytokinesis, and cell migration (*4*, *5*). The physical properties of the cell cortex additionally depend on the architecture and density of actin-filament networks generated by distinct actin-assembly factors (*2*, *6*). In cells, actin polymerization is mostly initiated by the actin-related proteins 2 and 3 (Arp2/3) complex and formins (*7*). The Arp2/3 complex creates branches at the side of preexisting mother filaments, generating a dense actin meshwork at the front of migrating cells, while formins nucleate and elongate long and linear actin filaments in distinct compartments (*7*, *8*).

A major subgroup of the formin family is composed of Diaphanous-related formins (DRFs), which are autoinhibited due to intramolecular interaction of regulatory domains (*9*) and activated by guanosine triphosphate (GTP)–bound Rho-family guanine nucleotide-binding proteins (guanosine triphosphatases) (*10*, *11*). Previous work has reported that most of the cortical actin in M2 melanoma and HeLa cells is generated by the Arp2/3 complex and the DRF mDia1 (DRF1) (*12*). Although only about 10% of the cortical F-actin in these cells is formin dependent, atomic force microscopy (AFM) measurements suggest that the formin-generated filaments are the major determinant regulating their macroscopic mechanical properties (*13*). Notwithstanding the important role of mDia1 in M2 and HeLa cells, it is currently unclear which of the 15 known mammalian formins regulate cortex architecture in other cell types. Current literature additionally suggests the involvement of the DRFs FH1/FH2 domain-containing protein 1 (FHOD1) (*12*), Disheveled-associated activator of morphogenesis 1 (Daam1) (*12*, *14*), mDia2 (DRF3) (*15*, *16*), and mDia3 (DRF2) (*17*, *18*), although endogenous mDia2 has recently been shown to localize mainly to the mitotic spindle and midbody of B16-F1, NIH 3T3, and HeLa cells (*19*).

In these previous studies, the functions and mechanical properties of the cell cortex were examined primarily in mitotic, rounded, blebbing, and largely immotile cells, raising the question whether these findings are generally applicable to polarized and migrating cells exhibiting a more complex actin architecture (*3*). Of note, cell migration is governed by polarization, adhesion strength, and cytoskeletal activities, which collectively result in the generation of site-specific forces (*20*, *21*). Because of substantial variations in these activities across different cell types, this process is further subdivided into amoeboid and mesenchymal migration, representing two extremes on a broad spectrum (*22*). Mesenchymal migration, characterized by slow motility, is defined by strong cell-matrix adhesion, the formation of prominent stress fibers (SFs), and the presence of a protruding lamellipodium at the leading edge (*23*). Conversely, amoeboid migration, as exemplified by neutrophils and *Dictyostelium* cells, is characterized by weak and transient adhesions, a rounded cell morphology, actin-rich protrusions or blebs at the front, and myosin-driven contractions at the rear (*24*). In the highly motile and weakly adherent *Dictyostelium* amoebae, the three DRFs ForA, ForE, and ForH are not uniformly distributed in the cell cortex but accumulate prominently at the rear of migrating cells to withstand high actomyosin-based contractility and guide the generated hydrostatic pressure to the front facilitating Arp2/3-driven protrusion and cell migration (*18*, *25*). Their combined loss has detrimental effects on cortex rigidity, cytokinesis, morphogenesis, and cell migration in two-dimensional (2D) confinement, but unexpectedly, it even slightly increases cell migration in unconfined settings (*18*). Notably, in the relatively fast mesenchymal B16-F1 mouse melanoma cells, which form mostly transient adhesions, active mDia1 and mDia3 are also prominently localized at the cell cortex of the trailing edge, but in this case, their combined loss reduced migration rate by almost 20%, suggesting that this may have been caused by their stronger adhesion compared to *Dictyostelium* amoeba (*18*).

Yet, whether and how cell cortex mechanics affects cell migration and mechanotransduction in highly adherent cells such as fibroblast is still largely elusive. Assuming that detachment of old FAs necessitates substantial actomyosin-generated forces of the cortex for trailing edge retraction for efficient migration, we reasoned that loss of cortical formins, yielding a functionally compromised cell cortex in these cells, will markedly perturb adhesion dynamics and cell migration. To experimentally test this hypothesis, we inactivated mDia1 and mDia3 by CRISPR-Cas9 technology in fibroblasts and systematically explored the impacts of a perturbed cell cortex on efficiency of 2D and 3D migration, adhesion, and integrity of the F-actin cytoskeleton. We found that the severe cell cortex defects in mDia1/3-deficient fibroblasts markedly affect relative migration rate as compared to less adhesive cell types. Notably, our study also reveals a profound reinforcement of matrix adhesion and contractility, resulting in highly stretched mutant cells that are prone to local rupture of SFs and the cell membrane, leading to the formation of cellular macroapertures distinct from the previously characterized transendothelial cell macroapertures (TEMs) (*26*). Thus, our work demonstrates the fundamental relevance of cortical formins for the resilience of tightly adherent and mechanically stressed cells.

## RESULTS

### Combined loss of mDia1/3 in highly adherent cells markedly impairs cell motility

**Fig. 1.**
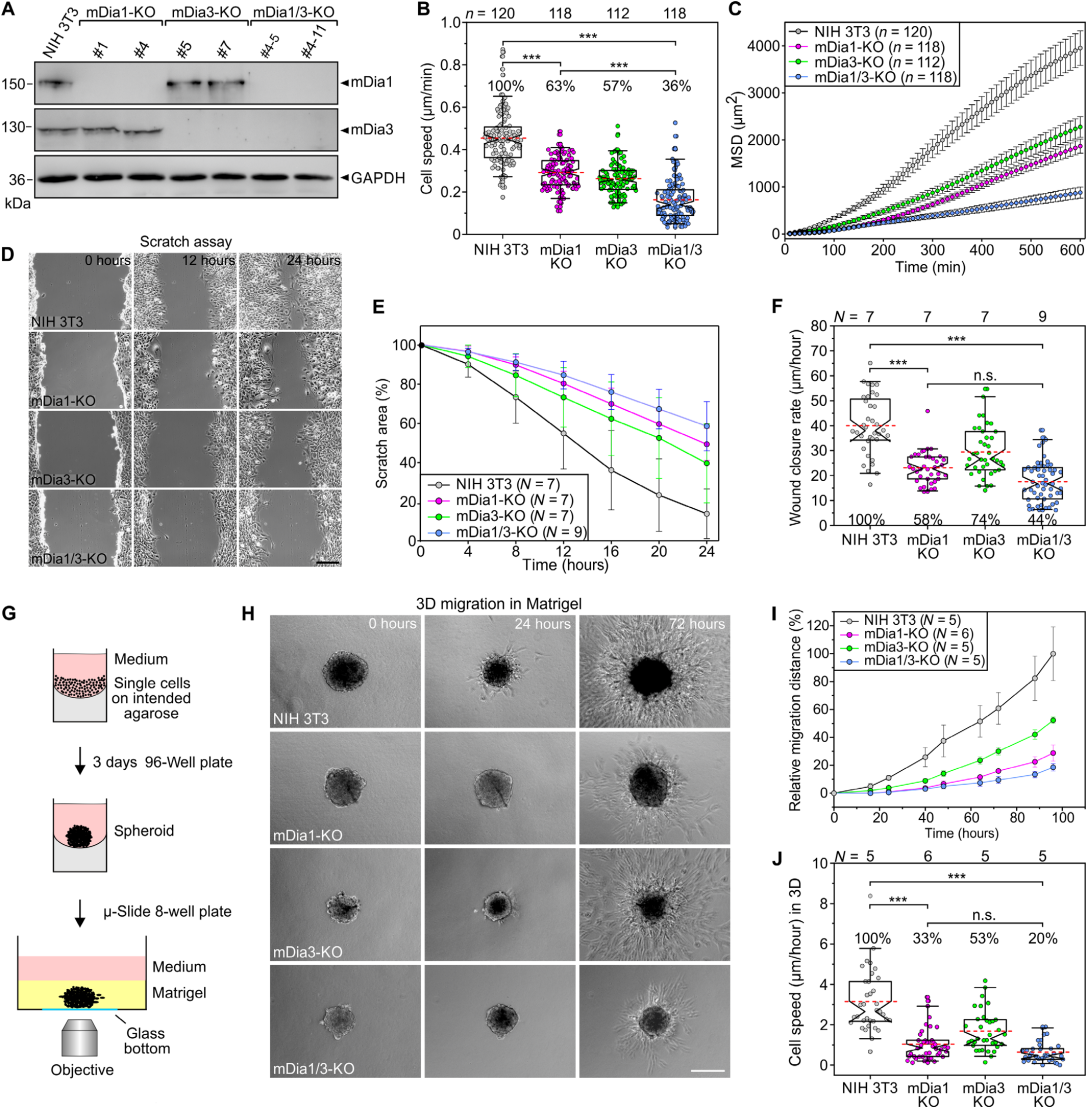
Loss of mDia1 and mDia3 impairs cell migration. (**A**) Loss of mDia1 and mDia3 by CRISPR-Cas9 in NIH 3T3 cells was confirmed by immunoblotting using specific antibodies. GAPDH was used as loading control. (**B**) Consecutive elimination of mDia1 and mDia3 increasingly decreased 2D cell migration on FN. (**C**) Analyses of mean square displacement of NIH 3T3 control versus mutant cells. Data represent means ± SEM. (**D**) Representative images from wound scratch assays of control and mutant cells as indicated. Scale bar, 200 μm. (**E**) Reduction of wound scratch area over time. Data indicate mean ± SD. (**F**) Quantification of wound closure rate. (**G**) Scheme of experimental setup for analysis of 3D cell migration. (**H**) Representative images from 3D migration movies of control and mutant cells in hydrogel. Scale bar, 200 μm. (**I**) Quantification of relative migration distance over time. (**J**) Quantification of cell speed in 3D. [(B), (F), and (J)] Boxes in box plots indicate 50% (25 to 75%) and whiskers 90% (5 to 95%) of all measurements, with dashed red lines depicting the means. Medians are highlighted by indentation of boxes. Kruskal-Wallis test with Dunn’s multiple comparison test. Percentages are shown to better illustrate the differences between cell lines. [(B) and (C)] *n*, number of cells. [(E), (F), (I), and (J)] *N*, number of movies analyzed. [(B), (C), (E), (F), (I), and (J)] Results are pooled data from at least four biologically independent replicates. ***P < 0.001. n.s., not significant.

To examine the function of the actin-rich cortex in highly adherent cells, we eliminated mDia1 and mDia3 by CRISPR-Cas9 technology individually or in combination in NIH 3T3 fibroblasts. Respective protein loss in independent clonal cell lines was confirmed by immunoblotting and sequencing of target sites (Fig. 1A and fig. S1). Migration rates of NIH 3T3 wild-type (control) and mutant cells on fibronectin (FN) were then analyzed by phase-contrast, time-lapse microscopy. Consecutive mDia member removal decreased 2D migration rate by an intermediate level in the single mDia-KO mutants and more strongly in the mDia1/3-KO mutant compared to control (Fig. 1B), supporting their functional synergy. Then, we calculated the mean square displacement (MSD) to assess their effective directional movement. All mDia mutants displayed incrementally decreasing and lower MSD levels compared to control, with the double mutant being almost nonmotile (Fig. 1C). Next, we analyzed directional cell migration in wound closure scratch assays (Fig. 1D). Average wound closure rates were reduced in the single-KO mutants, but again most strongly in the mDia1/3-KO cells compared to control (Fig. 1, E and F). Last, we analyzed migration in a 3D setting by monitoring the dissemination of cells from spheroids embedded in hydrogel (Fig. 1, G and H). Notably, 3D migration of mDia1/3-deficient fibroblasts was decreased by approximately 80% compared to control, with the single-KO mutants exhibiting intermediate cell speeds (Fig. 1, I and J). Thus, in contrast to less adherent cell types (*18*), cortical-formin removal caused a profound defect in cell migration in highly adherent NIH 3T3 cells.

### Adherent mDia1/3-KO fibroblasts compensate impaired cortical function by increased cortical tension

**Fig. 2.**
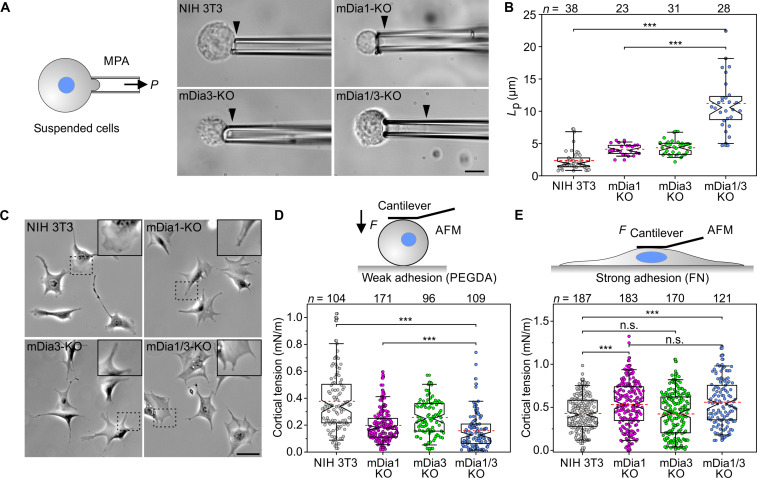
Elimination of mDia1 and mDia3 disrupts the contractile actin cortex. (**A**) Schematic and representative images from micropipette aspiration assays illustrating the projection lengths of probed cells (black arrowheads) as indicated. Scale bar, 10 μm. (**B**) Initial indentation length (*L*_p_) of control and mutant cells, as indicated, was determined by micropipette aspiration using a constant suction pressure of 50 Pa from time-lapse movies; data correspond to movie S1. (**C**) Representative phase-contrast images of control and mutant cells migrating on FN. Insets, enlarged images of dashed boxed regions depicting cell protrusions. Scale bar, 50 μm. (**D** and **E**) Schematic and quantitative analysis of cortical tension by AFM of control and mutant cells that adhere weakly or strongly to the underlying substrate. Note increased cortical tension of mDia1/3-KO cells on FN as compared to NIH 3T3 control. [(B), (D), and (E)] Boxes in box plots indicate 50% (25 to 75%) and whiskers 90% (5 to 95%) of all measurements, with dashed red lines depicting the means. Medians are highlighted by indentation of boxes. Kruskal-Wallis test with Dunn’s multiple comparison test. Results are pooled data from at least three biologically independent replicates. *n*, number of analyzed cells. ***P < 0.001. n.s., not significant.

To assess the viscoelastic properties of control and mutant cells, we performed micropipette aspiration assays (MPAs) of suspended cells to directly measure their cortical rigidity using a constant suction pressure of 50 Pa (Fig. 2A). Initial indentation lengths (*L*_p_) were already longer in single-KO mutants and were markedly increased in mDia1/3-KO double-mutant cells compared to control, indicative of a severely perturbed cell cortex (Fig. 2B and movie S1). Accordingly, phase-contrast imaging of control and mDia1/3-KO fibroblasts revealed notable differences in cell morphology (Fig. 2C). In contrast to the well-polarized NIH 3T3 cells, which displayed broad lamellipodia, the considerably larger double-mutant cells were unpolarized, displayed a hyperstretched spiderweb-like phenotype, and were considerably flatter as evidenced by the absence of halos and as assessed by calculation of the ratio between the cell volume and area (Fig. 2C and fig. S2). Moreover, the broad lamellipodia were largely absent and replaced by rod-like structures protruding from the cell periphery (*25*).

Next, we used AFM to derive cortical tension from force-compression and relaxation experiments. To compare the contributions of cell-substrate adhesion and friction, cells either weakly adhering to polyethylene glycol diacrylate (PEGDA) or strongly adhering to FN were probed with a tipless AFM cantilever. Consistent with the MPA measurements, cortical tension of cells on PEGDA was reduced by about 58% upon combined loss of both formins whereas individual loss of mDia1 and mDia3 resulted in intermediate reductions (Fig. 2D). Unexpectedly, in adhering double-mutant cells, cortical tension was increased by more than 30% compared to control (Fig. 2E), suggesting activation of compensatory mechanisms to mimic cortical integrity. As increased cortical tension is required for mitotic cell rounding and cytokinesis (*27*), we then examined control and mDia1/3-KO cells during mitosis. In sharp contrast to control and adherent mutant cells, mitotic mDia1/3-KO cells exhibited extensive and continuous blebbing, supporting the notion of a dysfunctional cell cortex, and took significantly longer to complete cytokinesis (fig. S3 and movie S2).

### Loss of mDia1/3 elicits the formation of amplified focal adhesions

**Fig. 3.**
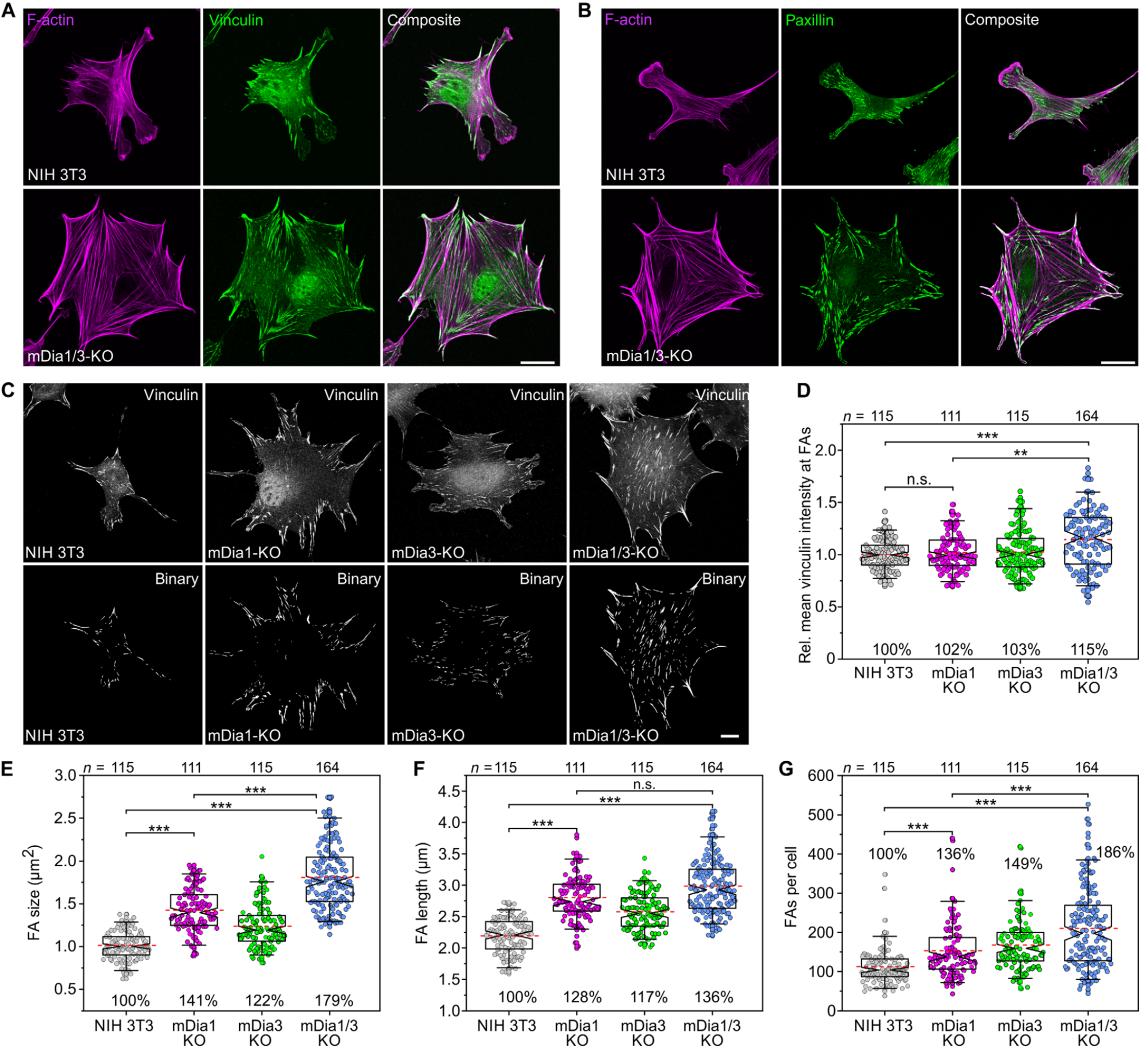
Loss of mDia1 and mDia3 promotes FA formation. (**A**) Representative images of control and mutant cells seeded on FN and stained for vinculin and the F-actin cytoskeleton. Scale bar, 20 μm. (**B**) Representative images of control and mutant cells seeded on FN and stained for paxillin and the F-actin cytoskeleton. Scale bar, 20 μm. (**C**) Representative micrographs of control and mutant cells stained for vinculin before (top) and after binarization (bottom). Scale bar, 10 μm. (**D**) Quantification of relative mean vinculin intensities in FA. (**E**) Quantification of FA size. (**F**) Quantification of FA length. (**G**) Quantification of FA number per cell. [(D) to (G)] Boxes in box plots indicate 50% (25 to 75%) and whiskers 90% (5 to 95%) of all measurements, with dashed red lines depicting the means. Medians are highlighted by indentation of boxes. Kruskal-Wallis test with Dunn’s multiple comparison test. *n*, number of cells. Results are pooled data from six biologically independent replicates. Percentages are shown to better illustrate the differences between cell lines. ***P < 0.001. n.s., not significant.

To examine whether loss of mDia1/3 affects cell-substrate adhesion, we first examined focal adhesions (FAs) and the F-actin cytoskeleton. Compared to control, in which FAs were evenly distributed, vinculin- and paxillin-labeled FAs of mDia1/3-KO cells were markedly enlarged and particularly prominent in the spiky protrusions at the cell periphery, and the cells traversed and were frequently contoured by exceptionally thick SFs (Fig. 3, A and B). The latter observation was rather unexpected, particularly because mDia1 has been implicated in SF formation (*28*, *29*). Thus, we used mass spectrometry to identify expressed actin nucleators in both control and mDia1/3-KO cells. In addition to the Arp2/3 complex, Cobl1, and several formins, we identified inverted formin-2 (INF2) and FHOD1, which are known to localize to SFs, and the latter also to drive their assembly (*30*, *31*) (fig. S4). Notably, FHOD1 was up-regulated by almost 50%, suggesting that this formin may be responsible for enhanced SF formation in mDia1/3-KO cells. Control and mutant cells stained for vinculin were then analyzed for various FA features (Fig. 3C). Vinculin intensity was only slightly elevated in both single mutants but increased by more than 15% in the mDia1/3-KO mutant compared to control (Fig. 3D). FA size in mDia1/3-KO cells was increased by 79% compared to control (Fig. 3E). Moreover, FA length and number increased by 36 and 86% in the double mutant compared to control (Fig. 3, F and G). This trend was also observed in mDia1/3-deficient CF-1 fibroblasts (figs. S1 and S5), confirming specificity of phenotype.

Increased membrane tension was shown to impair lamellipodial protrusion (*20*). Given the stretched morphology of mDia1/3-KO cells, we compared lamellipodial parameters in control and mutant cells by staining for the lamellipodial marker proteins Wiskott-Aldrich syndrome protein–family verprolin-homologous protein 2 (WAVE2) (*32*) and vasodilator-stimulated phosphoprotein (VASP) (*33*) together with phalloidin. Consistent with previous work in *Dictyostelium* and B16-F1 mouse melanoma cells (*18*), combined loss of mDia1/3 in fibroblasts led to the formation of multiple protrusive fronts (fig. S6, A and D). However, unlike the former, which form prominent protrusions in the absence of cortical formins (*18*), the highly adherent mDia1/3-deficient fibroblasts only formed sparse, rod-like lamellipodia emanating from spiky cell projections. Notably, their length and width, as well as the VASP, WAVE2, and F-actin intensities in lamellipodia, were significantly decreased compared to control (fig. S5, B, C, and E to G), supporting the idea of high surface tension in mDia1/3-KO cells. Conversely, cell spreading rate and WAVE2 intensity at the lamellipodium tip of spreading mDia1/3-KO cells were markedly increased compared to control (fig. S7, A to E). Last, we visualized the development of the spiderweb-like phenotype by time-lapse imaging and found an initial formation of numerous, multidirectional fronts along the periphery that expanded the cells and eventually stopped protruding, likely due to increased tension counteracting actin-based protrusion, and subsequently stabilized by increased adhesion (fig. S7F and movie S3).

### Loss of mDia1/3 impairs FA assembly and turnover

**Fig. 4.**
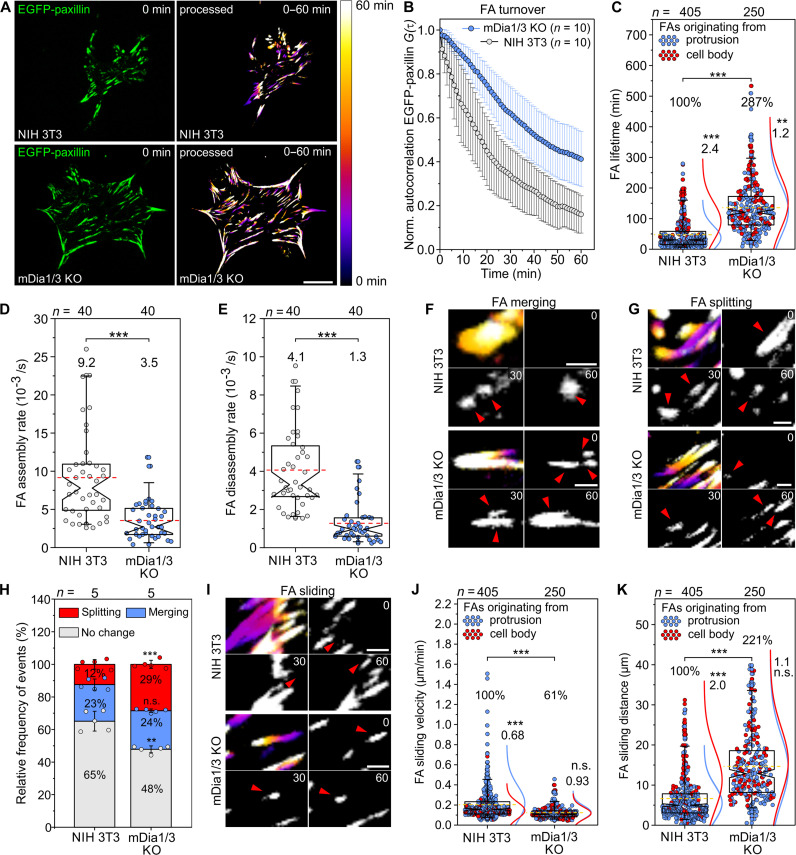
Loss of mDia1 and mDia3 affects FA turnover and dynamics. (**A**) Representative images from time-lapse, TIRF movies of control and mDia1/3-KO cells migrating on FN and displaying EGFP-paxillin accumulation in FAs (left); data correspond to movie S2. Temporal color-coded time stacks of the same cells over a 60-min period (right). Bar on far right shows color-coded time stamp. Scale bar, 25 μm. (**B**) Quantification of FA turnover. Normalized autocorrelation of EGFP-paxillin fluorescence. (**C**) Quantification of FA lifetime originating from protrusions or the cell body. (**D** and **E**) Quantification of FA assembly and disassembly rates. (**F** and **G**) Representative examples of merging and splitting FAs in control and mDia1/3-KO cells color coded as in (A). Time is indicated in minutes. Scale bars, 3 μm. (**H**) Quantification of FA splitting and merging. Boxes indicate mean ± SD. Student’s *t* test. (**I**) Representative examples of sliding FAs in control and mDia1/3-KO cells color coded as in (A). Time is indicated in minutes. Scale bars, 3 μm. (**J** and **K**) Quantification of FA sliding velocity and distance. [(B) and (H)] *n*, number of cells. [(C), (D), (E), (J), and (K)] *n*, number of FAs. Boxes in box plots indicate 50% (25 to 75%) and whiskers 90% (5 to 95%) of all measurements, with dashed red lines depicting the means. Medians are highlighted by indentation of boxes. Mann-Whitney *U* test. Results are pooled data from six biologically independent replicates. Percentages or calculated values are shown to better illustrate the differences between cell lines. [(C), (J), and (K)] Blue and red curves depict normal distribution curves of data points in box plots, corresponding to FAs originating from protrusions or the cell body, respectively. The values above the curves correspond to the respective ratios of lifetimes, sliding velocity or sliding distance of the FA populations originating from the cell body and lamellipodium. [(C), (D), (E), (H), (J), and (K)] **P < 0.01 and ***P < 0.001. n.s., not significant.

To unravel the molecular basis for enhanced FA size, we transfected control and mDia1/3-KO cells with enhanced green fluorescent protein (EGFP) fused to paxillin and examined adhesions by time-lapse, total internal reflection fluorescence (TIRF) imaging to follow their dynamics during random migration on FN (movie S4). Temporal color-coded images, enabling comparison of FAs in cells over time, indicated that FAs of the double mutants were considerably more stable than FAs of control cells (Fig. 4A). Autocorrelation analysis of EGFP-paxillin fluorescence revealed that FA turnover was substantially slower in mDia1/3-KO cells compared to control (Fig. 4B). Detailed FA turnover quantification revealed a ~2.9-fold longer FA lifetime on average in the double mutant compared to control (Fig. 4C). We found that in control cells, FAs emanating from cell protrusions disassembled more rapidly than FAs formed in the cell body. In contrast, in mDia1/3-KO mutants, FAs were generally not dissolved upon lamellipodial retraction, but relocated into the cell interior and subsequently disassembled with similar dynamics as FAs formed in the cell body. We then inferred assembly and disassembly rates of FAs by tracking EGFP-paxillin fluorescence of individual FAs over time (*34*). Notably, both assembly and disassembly rates were decreased in mDia1/3-KO cells (Fig. 4, D and E, and fig. S8). However, because the assembly phase is relatively short compared to the steady state and disassembly phase, the slower FA assembly is negligible in terms of lifetime. Quantification of changes in FA merging and splitting showed that merging was unchanged, but FA splitting was significantly increased in Dia1/3-KO cells compared to control (Fig. 4, F to H). Notably, all examined FAs showed centripetal sliding (Fig. 4I). In mDia1/3-KO cells, the sliding velocity of FA was decreased by almost 40%, but due to their longer lifetime, the sliding distance was increased almost threefold (Fig. 4, J and K). Combined, these data demonstrate that loss of mDia1/3 markedly impairs FA dynamics, resulting in much larger and more stable FAs.

### Combined loss of mDia1/3 alters F-actin architecture

**Fig. 5.**
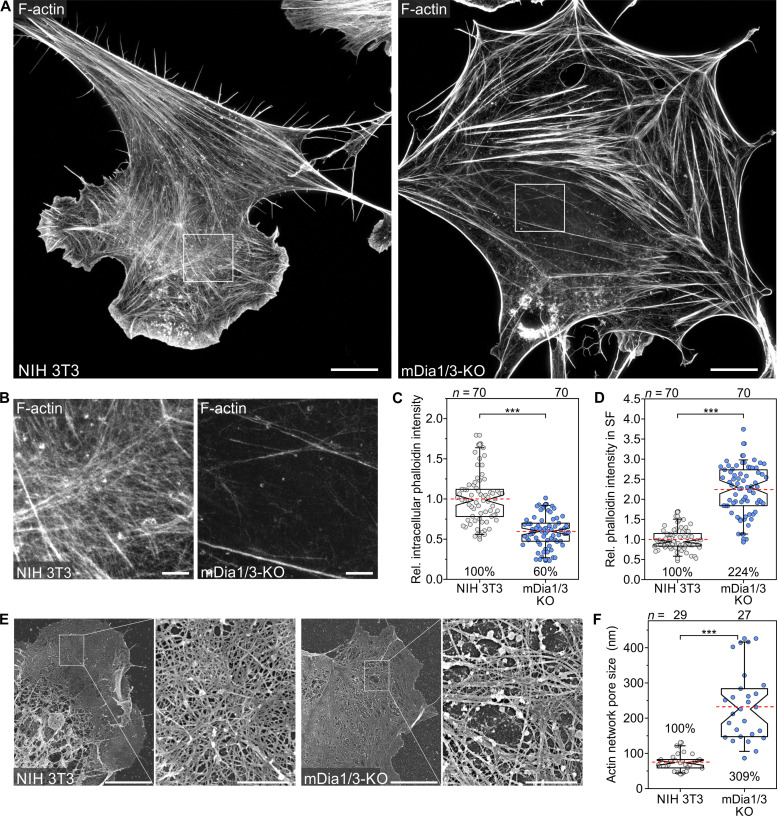
mDia1/3-deficient fibroblasts exhibit marked changes in F-actin cytoskeleton architecture. (**A**) Representative STED images of an NIH 3T3 control and mDia1/3-KO cell stained with STAR635-phalloidin for the F-actin cytoskeleton. Scale bars, 10 μm. (**B**) Enlarged image of boxed regions shown in (A). Scale bar, 1.5 μm. (**C**) Quantification of relative phalloidin intensity in regions lacking SFs mainly representing the cortical F-actin cytoskeleton. (**D**) Quantification of relative phalloidin intensity in SFs. (**E**) Representative SEM images of ultrastructural cortex architecture in a control and an mDia1/3-KO cell after detergent extraction. Enlarged images of boxed regions are shown on the right. Scale bars, 5 μm at low magnification or 1 μm at higher magnification. (**F**) Quantification of cortical actin network pore size. [(C), (D), and (F)] Boxes in box plots indicate 50% (25 to 75%) and whiskers 90% (5 to 95%) of all measurements, with dashed red lines depicting the means. Medians are highlighted by indentation of boxes. Mann-Whitney *U* test. Results are pooled data from at least three biologically independent replicates. Percentages are shown to better illustrate the differences between cell lines. *n*, number of cells analyzed. ***P < 0.001.

To explore the consequences of mDia1/3 loss on F-actin architecture at the nanoscale, we used 2D stimulated emission depletion (STED) superresolution imaging of fixed cells. In contrast to control cells, which displayed prominent lamellipodia and a dense actin meshwork interspersed with many thin SFs, the double mutants formed fewer, but exceptionally thick SFs at the expense of a dense filament meshwork in other areas of the cell, presumably representing the cortical F-actin cytoskeleton (Fig. 5A and fig. S9). Consistently, quantification of phalloidin intensities in sectors devoid of SFs revealed a reduction of F-actin content by more than 40% in mDia1/3-KO cells compared to control (Fig. 5, B and C). Conversely, the fluorescence intensity in SFs was more than doubled (Fig. 5D), indicating increased contractility of the SF system in mDia1/3-KO cells. To exclude potential extraction artifacts, we confirmed these findings by live-cell STED imaging (fig. S10). Last, we explored the ultrastructural cortex architecture by scanning electron microscopy (SEM) after detergent extraction of cells. In contrast to the dense, homogeneous cortical meshwork in control cells with a mean pore size of approximately 75 nm (Fig. 5, E and F), combined loss of mDia1/3 led to substantial changes in cortical actin organization, with generally less densely packed filaments with a mean pore size of about 230 nm interspersed with large pores up to 450 nm.

### Amplified FAs correlate with higher mechanical load and increased adhesion

**Fig. 6.**
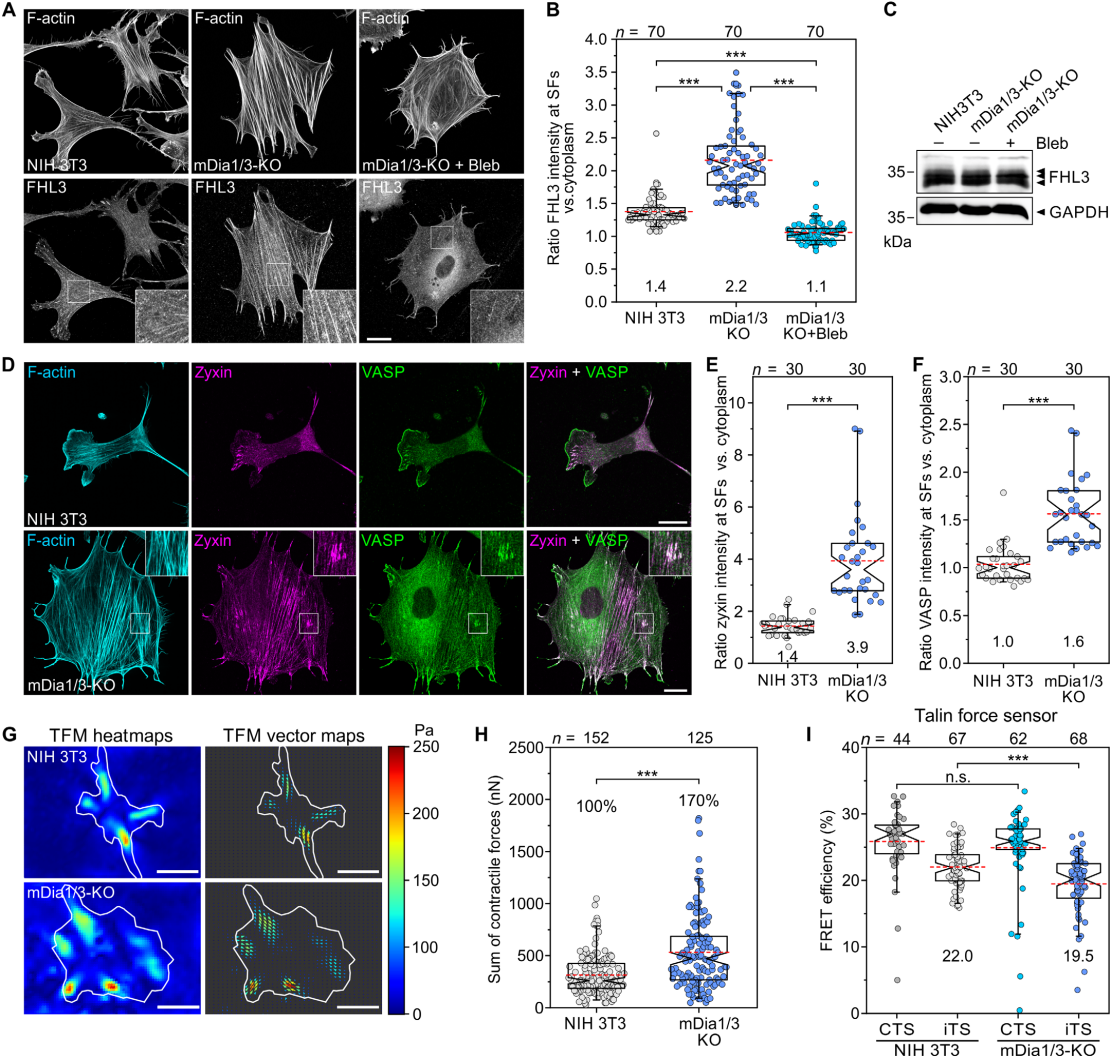
Loss of mDia1 and mDia3 amplifies force development. (**A**) Representative images of an NIH 3T3 control and an mDia1/3-KO cell on FN treated with 50 μM blebbistatin (bleb) for 30 min as indicated and stained for the four and a half LIM domains 3 protein (FHL3) and phalloidin. Insets, enlarged images of boxed regions. Scale bar, 20 μm. (**B**) Quantification of FHL3 intensity at SFs in the absence or presence of blebbistatin. Kruskal-Wallis test with Dunn’s multiple comparison test. Results are pooled data from three biologically independent replicates. (**C**) Comparable expression of FHL3 in control and mDia1/3-KO cells was confirmed by immunoblotting using FHL3-specific antibodies. GAPDH was used as loading control. (**D**) Representative images of a fixed control and an mDia1/3-KO cell on FN costained for the F-actin cytoskeleton, zyxin and VASP. Insets, enlarged images of boxed regions depicting an SF damage site. Scale bar, 20 μm. (**E**) Quantification of zyxin intensity on intact SFs. (**F**) Quantification of VASP intensity on intact SFs. (**G**) Images depicting heat map and traction force field representations of cells indicated. Scale bar, 10 μm. Force scale bar is in pascals and arrows represent the local force magnitude and orientation. (**H**) Quantification of contractile forces. (**I**) Quantification of FRET efficiencies in control and mDia1/3-KO mutants expressing the internal talin tension sensor (iTS) or the C-terminal no-force control talin probe (CTS). [(B), (E), (F), (H), and (I)] Boxes in box plots indicate 50% (25 to 75%) and whiskers 90% (5 to 95%) of all measurements, with dashed red lines depicting the means. Medians are highlighted by indentation of boxes. Mean values and percentages are shown to better illustrate the differences between cell lines. *n*, number of cells analyzed. [(E), (F), (H), and (I)] Mann-Whitney *U* test. Results are pooled data from at least three biologically independent replicates. ***P < 0.001. n.s., not significant.

Mechanical force is well known to play a role in regulating FA size (*35*), at least during the initial maturation phase (*36*). Similarly, SFs have also been shown to undergo reinforcement in response to strain (*37*). Because the highly stretched cell shape, together with the exaggerated FAs and thick SFs, all pointed to high contractility and mechanical tension of the FA/SF system in mutant cells, we next compared force-activated binding of the actin-filament tension sensor four-and-a-half LIM domains 3 (FHL3) (*38*) to SFs in fixed control and mDia1/3 KO cells (Fig. 6A). In contrast to control, where FHL3 was moderately enriched at SFs, in the mDia1/3-KO mutant, FHL3 binding to SFs was markedly increased by almost 60%, despite comparable FHL3 expression (Fig. 6, B and C). Consistently, decreasing actomyosin contractility in the double mutant with the myosin 2-inhibitor blebbistatin (*39*) virtually abolished FHL3 binding, confirming increased SF strain. Consistently, and in contrast to equal amounts of nonmuscle myosin 2A (NM2A), we found an approximately 40% increase in phosphorylated myosin light chain 2 (pMLC2) accumulation at SFs in the mutant compared to control (fig. S11, A to D). However, despite increased SF contractility and tension in mDia1/3-KO cells, the overall SF architecture was not affected by loss of mDia1/3, as evidenced by unchanged intensity and distribution of α-actinin-1 at SFs (fig. S11, E to I).

In agreement with previous work showing that strain induces local zyxin-dependent VASP recruitment to damaged SFs (*40*), we observed strong accumulation of zyxin and VASP at putative damage sites, but additionally a continuous localization along SFs in the double mutant (Fig. 6, D to F). Quantification of SF strain sites on substrates of varying stiffness (fig. S12) additionally revealed multiple strain sites per cell in the double mutant, with the number decreasing progressively as stiffness declined. In contrast, only a small fraction of control cells formed a single strain site, which was observed exclusively on stiff substrates. Because FAs function as sites of force transmission between the cytoskeleton and the extracellular matrix (ECM) (*41*), we then compared traction forces exerted by cells to the ECM. Albeit traction stress was similar when normalized to total cell or FA area, the sum of contractile forces was increased by almost 70% in double-mutant cells compared to control (Fig. 6, G and H), demonstrating increased force transmission to the substrate.

To obtain molecular-scale information on force transduction, we lastly used a single-molecule calibrated Förster resonance energy transfer (FRET)–based tension sensor based on the integrin activator talin-1, which is critical for force transduction in FAs (*42*). Quantification of FRET efficiencies by fluorescence lifetime imaging microscopy (FLIM) in cells expressing a control or the talin force sensor revealed increased mechanical tension on talin-1 in mDia1/3-KO cells compared to control (Fig. 6I). As FLIM-FRET experiments utilized the FL-based tension sensor module, which is characterized by an almost digital force-FRET response, the data indicated that an increased number of talin-1 molecules were exposed to mechanical forces of at least 3 to 5 pN in mDia1/3-KO cells.

### Tension and contractility induce the formation of transcellular macroapertures in mDia1/3-KO mutants

**Fig. 7.**
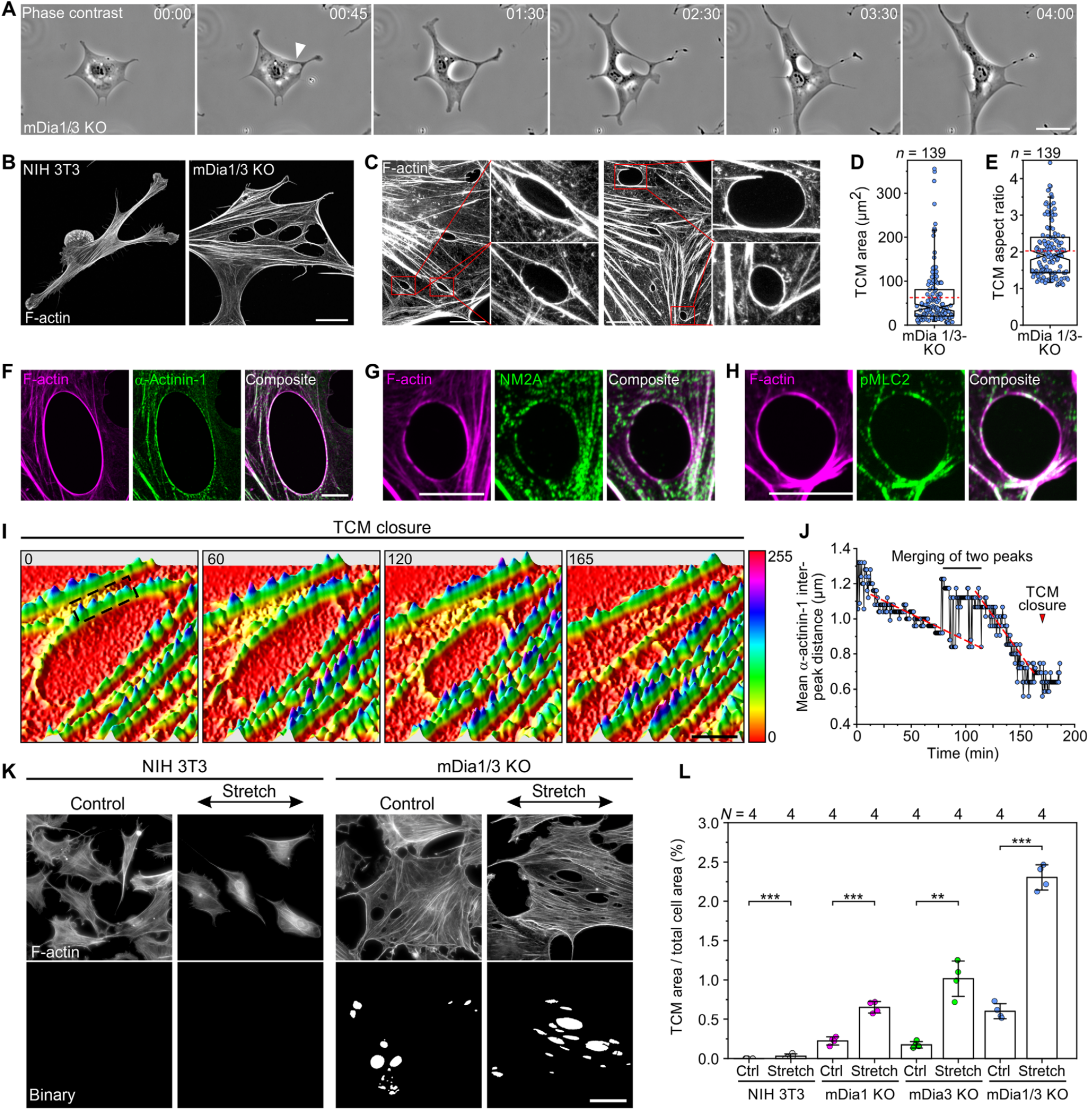
mDia1/3-deficient fibroblasts form tension and contractility-induced macroapertures. (**A**) Still images from a time-lapse, phase-contrast movie of an mDia1/3-KO cell migrating on FN illustrating the formation and aperture-like closure of a TCM; data correspond to movie S5. Scale bar, 50 μm. Time is indicated in hours and minutes. (**B**) Representative confocal z-stack projections of a control and an mDia1/3-KO cell forming multiple TCMs stained with phalloidin. Scale bar, 20 μm. (**C**) Close-up STED images of representative TCMs in mDia1/3-KO mutants stained with phalloidin. Insets, enlarged images of boxed regions. Scale bar, 10 μm. (**D**) Quantification of TCM size in mDia1/3-KO cells. (**E**) Quantification of TCM aspect ratio in mDia1/3-KO cells. (**F** to **H**) Representative confocal z-stack projections of TCMs stained with phalloidin and costained for α-actinin-1, nonmuscle myosin 2A (NM2A) or phosphorylated myosin light chain 2 (pMLC2). Scale bars, 10 μm. (**I**) Still images from a time-lapse TIRF movie illustrating TCM closure in an mDia1/3-KO cell expressing EGFP-tagged α-actinin-1; data correspond to movie S6. (**J**) Analysis of TCM closure in boxed, dashed region depicted in (I). Red dashed lines represent linear fits of data points. Red arrowhead indicates completion of TCM closure. (**K**) Representative epifluorescence and binarized images of unstretched and stretched control and mDia1/3-KO cells on elastic substrate derivatized with FN after phalloidin staining. Scale bar, 50 μm. (**L**) Quantification of TCM area versus total cell area in stretched and unstretched cells. Each data point represents the mean of an independent experiment in which at least 10 images were analyzed. Student’s *t* test. *N*, number of independent biological replicates. [(D) and (E)] Boxes in box plots indicate 50% (25 to 75%) and whiskers 90% (5 to 95%) of all measurements, with dashed red lines depicting the means. Medians are highlighted by indentation of boxes. *n*, number of cells analyzed. **P < 0.01 and ***P < 0.001.

In agreement with the severe cortical defects associated with increased mechanical load and adhesion, time-lapse imaging of mDia1/3-KO cells additionally revealed the presence of dynamic transcellular macroapertures (Fig. 7A and movie S5), resembling TEMs, evoked by bacterial Rho-inhibitory toxins (*26*). The macroapertures were absent in control cells, but found in mDia single mutants, albeit less frequently, and they vanished upon re-expression of full-length mDia1 or mDia3, confirming specificity of phenotype (fig. S13, A to D). Moreover, unlike the circular TEMs that are surrounded by a continuous F-actin ring (*43*, *44*), the considerably larger and elliptic macroapertures of mDia1/3-KO cells mostly displayed an uneven actin accumulation at their periphery (Fig. 7, B to E). These findings were again confirmed in mDia1/3-deficient mouse CF-1 fibroblasts (fig. S5). The macroapertures in the double mutant were mostly found in regions with low F-actin density and were commonly sandwiched between adjacent SFs (Fig. 7, B and C). Furthermore, in contrast to TEMs, which close by extension of lamellipodia-like structures into the empty space (*43*, *44*), the macroapertures of mDia1/3-KO mutants closed by constriction (movie S5). Consistently, they lacked the lamellipodial markers WAVE2 and VASP at their periphery (fig. S13E) but accumulated components of the contractile machinery such as α-actinin-1, NM2A, and pMLC2 in a striated pattern, resembling contractile SFs (Fig. 7, F to H).

We then used TIRF imaging of mDia1/3-KO cells expressing EGFP-α-actinin-1 and found that shortening of the sarcomere-like subunits at the macroaperture periphery coincides with closure (Fig. 7, I and J, and movie S6), strongly suggesting that this process is driven by actomyosin-based contraction. This was confirmed by blebbistatin treatment of mDia1/3-KO cells and revealed conversion of the elliptic macroapertures into circular TEM-like structures with a pronounced actin ring, as evidenced by morphology and invasion of lamellipodia-like structures containing VASP and WAVE2 into the void space (figs. S13, F to I, and S14 and movie S7). In addition, the fraction of ruptured macroapertures increased, demonstrating that actomyosin contractility within the actin ring counteracts the high tension in the mutant (figs. S13F and S14A). Notably, treatment with the Arp2/3 complex inhibitor CK666 resulted in fewer macroapertures, demonstrating that Arp2/3 is dispensable for closure (fig. S13, F and G). Consistently, treatment with both blebbistatin and CK666 restored macroapertures, confirming that closure of TEM-like structures induced by blebbistatin requires Arp2/3-dependent actin assembly. Thus, owing to the notable differences to TEMs, we designated the holes in mDia1/3-deficient fibroblasts as tension- and contractility-induced cellular macroapertures (TCMs). Last, to test whether TCMs can be induced by shear forces, control and mutant cells were subjected to mechanical strain on a uniaxial cell stretcher. After cyclic stretching by 20%, all mDia-deficient mutants, and especially mDia1/3-KO cells, showed a strong induction of TCMs compared to control (Fig. 7, K and L), confirming this view.

### SF rupture precedes TCM formation and is followed by enhanced protrusion

**Fig. 8.**
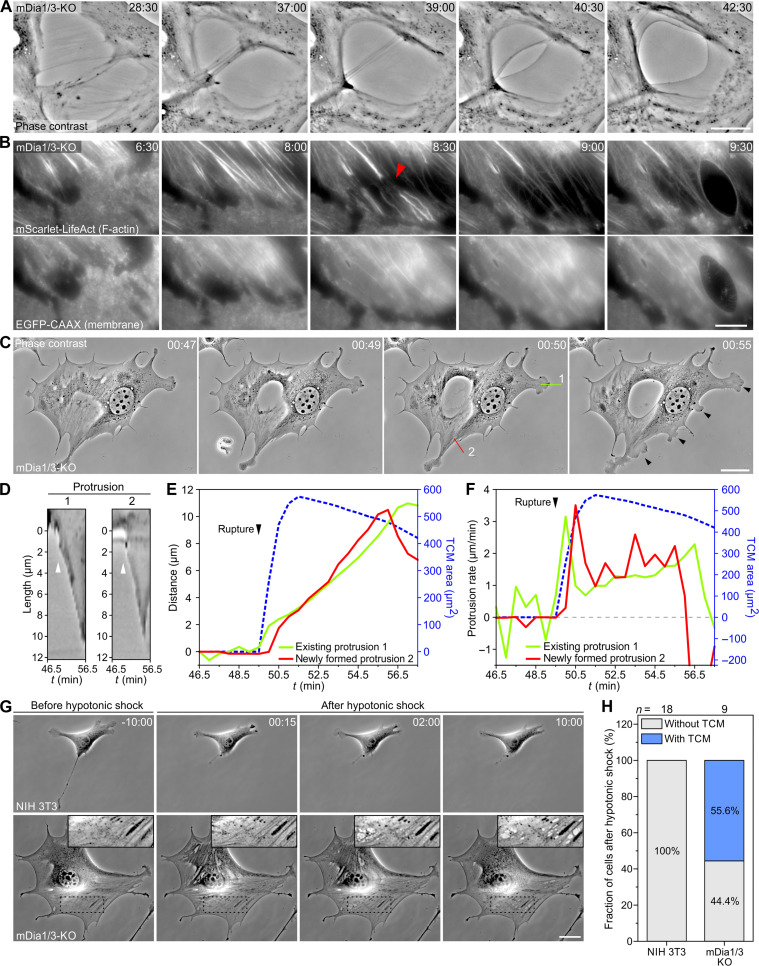
TCM formation is preceded by SF rupture and promotes lamellipodial protrusion. (**A**) Close-up images from a time-lapse, phase-contrast movie of an mDia1/3-KO cell on FN; data correspond to movie S8. Scale bar, 20 μm. Time is indicated in minutes and seconds. (**B**) Close-up images from a time-lapse TIRF movie of an mDia1/3-KO cell on FN expressing mScarlet-LifeAct (top) and EGFP-CAAX (bottom) showing the rupture of prominent SFs as indicated by the red arrowhead, followed by local expansion of this area and subsequent rupture of the plasma membrane leading to TCM formation; data correspond to movie S9. Scale bar, 20 μm. Time is indicated in minutes and seconds. (**C**) Still images from a time-lapse, phase-contrast movie of an mDia1/3-KO cell on FN exemplifying that formation of a prominent TCM is followed by a burst of protruding lamellipodia; data correspond to movie S10. Scale bar, 20 μm. Time is indicated in hours and minutes. (**D**) Kymographs along the two depicted lines as marked in (C) are shown. Arrowheads illustrate an increase in lamellipodial protrusion following the formation of the TCM. (**E**) Quantification of distance traveled by lamellipodia (green and red lines) versus TCM area (blue dashed line) over time. (**F**) Quantification of lamellipodial protrusion rates versus TCM area over time. Note marked decrease in protrusion rate shortly after the TCM begins to close again. (**G**) Still images from representative time-lapse, phase-contrast movies of a control and an mDia1/3-KO cell illustrating TCM formation after hypotonic shock exclusively in the mutant cell; data correspond to movie S11. Scale bar, 25 μm. Time is indicated in minutes and seconds. (**H**) Quantification of the fraction of cells forming TCMs directly after hypotonic shock. Boxes indicate fraction of cells. *n*, number of cells. Results are pooled data from three biologically independent replicates.

Time-lapse imaging of mDia1/3-KO cells at higher magnification revealed extensive pulling forces on the cytoskeleton before TCM formation (Fig. 8A and movie S8), suggesting that loosening or even rupture of SFs may precede initiation of TCMs. To experimentally test this idea, we used TIRF imaging of mDia1/3-KO cells coexpressing mScarlet-LifeAct and EGFP-CAAX to visualize the F-actin cytoskeleton and the membrane in live cells. Notably, we regularly observed rupture of prominent SF bundles followed by local expansion of this area, leading to temporally delayed membrane rupture and TCM formation (Fig. 8B and movie S9). As high membrane tension was shown to diminish actin-based protrusion (*20*, *45*), we eventually analyzed the consequences of TCM formation on lamellipodia protrusion. To this end, we recorded randomly migrating mDia1/3-KO cells and determined protrusion rates of existing and emerging lamellipodia instantly after TCM formation by kymograph analysis (Fig. 8, C and D, and movie S10). Notably, immediately after TCM formation, we observed a burst of persistently protruding lamellipodia. Their protrusion rate was fastest immediately after TCM formation and then gradually decreased upon TCM closure (Fig. 8, E and F), supporting the idea of impaired lamellipodial protrusion due to increased membrane tension. Last, to experimentally test whether membrane tension drives TCM formation, we exposed cells to hypotonic shock, which is known to rapidly increase membrane tension (*46*, *47*). Notably, numerous macroapertures were induced in mDia1/3-KO cells after the shock, but not in control cells (Fig. 8G and movie S11), corroborating the view that increased membrane tension promotes TCM formation.

## DISCUSSION

Cell migration of highly adherent cells such as fibroblasts faces multiple challenges in maintaining an intact cytoskeletal architecture, forming protrusions at the front and detaching at the rear, while simultaneously withstanding substantial intracellular and external forces in the presence of strong friction against the ECM (*23*). By using a multilevel approach, here we unravel the decisive role of the synergistically acting formins mDia1/3 in cell cortex mechanics and migration in fibroblasts. Contrary to poorly or moderately adherent cells forming rather weak and transient matrix adhesions (*18*), we found that concurrent loss of mDia1/3 in NIH 3T3 cells, associated with substantial cell cortex defects, severely impaired cell migration. Efficient cell migration requires the deadhesion of old FAs to allow retraction of the rear and is assumed to be primarily mediated through tyrosine phosphorylation and changes in SF tension (*48*, *49*). Depletion of mDia1 in rat C6 glioma cells was also reported to impair polarization, migration, and adhesion turnover, but not FA formation per se (*50*). Notably, this earlier study did not link mDia1 function to cortex mechanics, but instead assumed that mDia1 depletion impairs accumulation of active Src at FAs and therefore attenuates c-Src–mediated p130Cas phosphorylation in adhesions, suggesting a signaling role for mDia1 acting upstream of the Src-Cas-Crk pathway that regulates FA turnover. Our results rather suggest that actomyosin-mediated contractility of an intact cell cortex, which requires the long actin filaments generated by formins (*13*), substantially contributes to the detachment of FAs in highly adherent cells, as evidenced by the excessive sliding of FAs in mDia1/3-KO cells without disassembly and exuberant blebbing during mitosis. Although these cells compensate for loss of cortical integrity by enhanced spreading to increase apparent cortical tension, the stretched cortex lacking formin-generated filaments is apparently unable to contract adequately and generate sufficient force to detach old FAs from the ECM. This also explains the smaller impact of cortical formins on cell migration in weakly or moderately adherent cells, where lower forces are expected to be required for detachment of old FAs.

Furthermore, we found that mDia1/3 loss results in a stretched morphology that coincides with a marked increase in larger FAs connected to unusually thick SFs that transmit amplified traction forces to the ECM by talin, raising questions about the mechanistic details underlying this phenotype. Several formins, including mDia1, have been proposed to promote SF assembly by controlling actin polymerization at FAs (*28*, *29*, *51*, *52*). However, except for endogenous FHOD1 and INF2 (*30*, *31*), their localization to these structures has not been conclusively confirmed. Notably, our mass spectrometry results revealed a substantial increase in FHOD1 levels, implying that this formin could be driving the enhanced SF assembly in mDia1/3-KO cells.

In mesenchymal cells, lamellipodia regularly exhibit repeated protrusion and retraction cycles, and it has been proposed that during each protrusion phase, the load on polymerizing actin filaments gradually increases due to elevated membrane tension resulting from the expanding cell area, ultimately leading to stalled protrusion and retraction (*20*, *53*). Our data show that spreading of mDia1/3-KO fibroblasts with numerous multidirectional fronts results in a spiderweb-like morphology that is subsequently stabilized by large, stable FAs beneath the spiky projections that prevent retraction to relieve tension as in wild-type cells, thereby maintaining the stretched phenotype. The notable phenotype of mDia1/3-KO fibroblasts closely resembles the shape of modeled and live cells adhering to a limited number of discrete adhesion sites (*54*, *55*), suggesting that the stretched cell morphology results from a balance between tension of the inwardly curved peripheral SFs and surface tension in the cortical plane. Accordingly, the large peripheral FAs of mDia1/3-KO cells presumably serve as major anchor points effectively counteracting the contractile forces, which may facilitate stretching of the perturbed cortex.

Localized strain induced by thinning and elongation within discrete regions of SFs leads to rapid accumulation of zyxin and VASP at sites of strain-induced SF damage followed by SF repair (*40*). Notwithstanding this, global shear stress was also reported to reinforce SFs via the zyxin-VASP axis (*53*, *56*). We show that SFs of mDia1/3-KO cells exhibit increased tension and contractility as evidenced by increased FHL3 binding and MLC2 phosphorylation. Unlike in the control, we found multiple strain sites in the double mutant, even on ECM substrates with lower stiffness, which is expected to decrease contractility, suggesting that the entire SF system of the double mutant is under high tensile stress even in environments of physiological rigidity. Consistently, we found recruitment of zyxin and VASP not only to damaged sites, but also along intact SFs, likely promoting the reinforcement of SFs in mDia1/3-KO cells through sustained actin assembly. Moreover, these cellular activities and the observed higher force on talin strongly indicate increased tension within individual actin filaments, although comparable traction stresses were observed when normalized to cell or FA area. This, in turn, suggests that FAs in double-mutant cells exhibit a lower area density of molecular attachment sites to the ECM compared to control. Loss of mDia1/3 in Sertoli cells also impaired the cortical F-actin meshwork, but induced abundant noncontractile F-actin bundles reminiscent of SFs (*17*). The cause of different SF properties is currently unclear but may be cell type specific and linked to the nonmotile behavior and distinct adhesion properties of Sertoli cells, as depletion of mDia1 in MEFs was reported to increase SF tension (*29*).

The stretched morphology of mDia1/3-KO cells, presumably linked to elevated membrane tension, hinders protrusion and culminates in the development of TCMs, which share similarities with TEMs, but are mechanistically distinct. Bacterial toxins like EDIN or C3-exoenzyme induce TEMs by inhibiting RhoA signaling (*26*, *43*), thus preventing both mDia and Rho-associated kinase pathway activation (*57*), leading to a global relaxation of actomyosin contractility and disruption of FAs. This is assumed to induce cell spreading and consequently a reduction of cell height followed by a dewetting process of the apical and basal membranes (*58*), which forms TEMs to a line tension maximum of the macroaperture contour limiting their size, and they close by extension of lamellipodia-like structures (*44*). Unlike TEMs, however, TCMs are formed in the presence of high SF contractility and strong adhesion, and they close through an aperture-like mechanism. Consistently, treatment with blebbistatin converts the elliptic TCMs into round TEM-like structures that subsequently close by protruding lamellipodia. Our data suggest that TCM nucleation occurs in areas of low cell height where the defective cell cortex is not sufficiently stabilized by SFs. Rupture and retraction of SFs, which are closely associated with cortical F-actin filaments (*59*), are expected to further destabilize this region by triggering a local expansion of the perturbed cell cortex. This apparently exceeds the stress limit of the membrane, forcing mDia1/3-KO cells to form TCMs to relieve tension and avoid uncontrolled rupture of the inextensible plasma membrane and cell death. A sudden drop of surface tension is supported by the instant emergence of lamellipodial protrusions at the cell periphery instantly after TCM formation. Conversely, increase of cortical tension by hypotonic stress instantly induced TCMs. The data therefore demonstrate the outstanding importance of cortical formins in the guidance and regulation of cellular forces in highly adherent and mechanically stressed cells.

Here, we thoroughly analyzed the consequences of mDia1/3 removal in NIH 3T3 fibroblasts and validated key findings in CF-1 fibroblasts. A limitation of our study arises from the presence of numerous known and potentially as yet uncharacterized cortical formins, each possibly expressed at varying levels across different fibroblasts and tissues. Therefore, we cannot predict at this point whether removal of mDia1 and mDia3 in other fibroblasts will result in an identical phenotype if cell cortex function is additionally safeguarded by other formins. Our mass spectrometry data showed an up-regulation of Daam1 and FMNL3, which may partially compensate for the loss of mDia1/3. However, this needs to be thoroughly addressed in future work. In any case, the conclusions drawn from our study should be applicable to broader combinations of cortical formins beyond mDia1 and mDia3, as the functionality of the cell cortex is primarily determined by the abundance of long formin-generated filaments within the cortical network (*13*).

In conclusion, here we reveal the critical role of cortical formins mDia1/3 in highly adherent fibroblasts, providing major insights into the mechanisms governing cell migration and mechanotransduction. This study paves the way for future studies analyzing the functions of cortical formins in various mechanically stressed cells as found in the lung or heart, or in pathophysiological conditions such as fibrosis (*60*).

## MATERIALS AND METHODS

### Experimental design

The aim of this study was to uncover the role of formins in cell cortex mechanics and cell migration in highly adherent cells. To this end, we eliminated mDia1 and mDia3 by CRISPR-Cas9 technology in fibroblasts and analyzed mutant cells by various cell biological, biophysical, and imaging techniques, including MPA, AFM, cell stretching experiments, TFM, and FLIM, as well as confocal, superresolution STED, and scanning EM imaging.

### Constructs

The full-length cDNA of murine α-actinin-1 (isoform 1, UniProt ID Q7TPR4) was amplified by PCR from an NIH 3T3 cDNA library and inserted into the Xho I–Asp^718^ sites of pEGFP-C1 (Clontech). pmScarlet-C1 LifeAct was generated by replacing sequences encoding EGFP in plasmid pEGFP-C1-LifeAct (*61*) with a corresponding sequence encoding mScarlet from plasmid pmScarlet-αTubulin-C1 (Addgene_85045) using the Age I and Bsr GI restriction sites. The DNA oligonucleotides 5′-GATCTTGCCTCATCTTGTGAG-3′ and 5′-GTACCTCACAAGATGAGGCAA-3′ encoding the C-terminal CAAX motif of murine RhoA were annealed and inserted into the Bgl II–Asp718I sites of pEGFP-C1 to obtain pEGFPC1-CAAX. Plasmids pEGFP-N1 paxillin (*62*), pEGFP-C1-mDia1-FL (*18*), and pEGFP-C1-mDia3-FL (*18*) have been described. For the generation of recombinant antigens, respective sequences encoding full-length murine FHL3 (isoform1, residues 1 to 289, UniProt ID Q9R059) and α-actinin-1 (isoform 1, residues 1 to 892) were amplified from an NIH 3T3 cDNA library and inserted into the Eco RI–Sal I or Sal I–Not I sites of plasmids pGEX-6P-1 and pGEX-6P-3 (GE Healthcare), respectively. Fidelity of generated plasmids was confirmed by sequencing. A complete list of primers used in the study is provided in table S1.

### Cell culture and transfection

Mouse embryonic NIH 3T3 fibroblasts (CRL-1658, CVCL_0594) and primary mouse embryonic CF-1 fibroblasts (SCRC-1040, CVCL_5251) were purchased from the American Type Culture Collection. CF-1 cells were immortalized by transfection with pSV40Tag encoding the SV40 large T antigen (*63*). Wild-type NIH 3T3 and CF-1 cells and derived cell lines were cultured at 37°C and 5% CO_2_ in high-glucose Dulbecco’s modified Eagle’s medium (DMEM; Lonza, 12-614F) supplemented with 10% fetal bovine serum (Biowest, S1810-500), 2 mM UltraGlutamine (Lonza, BE17605EU1), and 1% penicillin-streptomycin (Biowest, L0022). NIH 3T3 cells and CF-1 cells were transfected with 3 or 1.5 μg of plasmid DNA, respectively, using JetPRIME transfection reagent (PolyPlus, 101000046) in six-well plates (Sarstedt, 83.3920) following the manufacturer’s instructions. Transfected cells were used 16 to 24 hours after transfection. Absence of mycoplasma in cell lines was routinely checked by the VenorGeM Mycoplasma Detection Kit (Sigma-Aldrich, MP0025). To inhibit myosin 2 contractility and Arp2/3 complex–mediated actin assembly, the cells were treated with 50 μM blebbistatin (Sigma-Aldrich, 203390) for 30 min or with 200 μM CK-666 (Sigma-Aldrich, SML0006) for 2 hours.

### Genome editing using CRISPR-Cas9

NIH 3T3 and CF-1 fibroblasts were transfected with plasmid pSpCas9(BB)-2A-Puro (PX459) V2.0 (Addgene_62988) carrying a single-guide RNA specifically targeting *DIAPH1* and *DIAPH2* encoding mDia1 and mDia3, respectively, as previously described (*18*). Twenty-four hours after transfection, cells were selected using puromycin (4 or 2 μg/ml; Invivogen, ant-pr-1) for 4 days, respectively. Subsequently, the cells were allowed to recover for at least 1 day in the absence of puromycin. For isolation of clonal knockout cell lines, single cells were seeded by visual inspection into 96-well microtiter plates and expanded in conditioned culture medium containing 40% sterile filtered NIH 3T3 or CF-1 medium taken from near-confluent plates. Clonal cell lines were analyzed for frameshifts in respective genes using the Tracking of Indels by Decomposition (TIDE) web tool (*64*) or sequencing of target sites. To this end, respective DNA fragments of about 700 bp encompassing the target sites were first amplified from genomic DNA using the primer pairs 5′-TGCAAGAAGGTAAAAGATTGGCT-3′ and 5′-CCGACAGACAAATGCCCATA-3′ for mDia1 or 5′-TGATTGCAGCTGCTTAGGG-3′ and 5′-CCTTAGCTATGAGTGGGCTCC-3′ for mDia3. Subsequently, the amplified fragments were inserted into pJet1.2 vector (Thermo Fisher Scientific, K1232) and used for transformation of *Escherichia coli* host XL-10 Gold (Agilent, 200315). At least 30 sequences were analyzed for each knockout clone. Knockouts were additionally verified by immunoblotting using specific antibodies.

### Protein purification

For expression of recombinant murine α-actinin-1 and FHL3 antigens, the *E. coli* strain Rossetta 2(DE3) (Novagen, 71400) was used. Expression of glutathione *S*-transferase (GST)–tagged fusion proteins was induced with 1 mM isopropyl-β-d-thiogalactoside (Carl Roth, 2316.4) at 21°C for 14 hours. The bacteria were harvested and lysed by ultrasonication in lysis buffer containing phosphate-buffered saline (PBS), pH 7.4, supplemented with 2 mM dithiothreitol (DTT; Carl Roth, 6908.2), 1 mM EDTA, 5 mM benzamidine (Sigma-Aldrich, 12072), 0.1 mM (4-[2-aminoethyl]benzenesulfonyl fluoride hydrochloride) (AEBSF) (AppliChem, A1421), Benzonase (Sigma-Aldrich, E8263, 1:1000), and 5% (v/v) glycerol (Carl Roth, 0798.3). Purification of respective proteins from bacterial extracts was performed by affinity chromatography using Protino glutathione-conjugated agarose 4B (Macherey-Nagel, 745500.100) following the instructions of the manufacturer. The GST-tag was removed by proteolytic cleavage with PreScission Protease (GE Healthcare, 27-0843-01), followed by a final polishing step of the proteins by size-exclusion chromatography on an Äkta Purifier System using either HiLoad 26/600 Superdex 200 or HiLoad 26/75 Superdex columns (GE Healthcare). Purified proteins were dialyzed against immunization buffer (150 mM NaCl, 1 mM DTT, and 20 mM tris-HCl, pH 7.4), stored in aliquots at −20°C, and were used for generation of polyclonal antibodies.

### Antibodies

The following primary antibodies were used in this study: mDia1 (*18*) (1:1000), mDia3 (*18*) (1:000), hVin1 (Sigma-Aldrich, V9131, AB_477629, 1:1000), paxillin (BD Biosciences, 610051, AB_397463 1:200), zyxin (Santa Cruz, sc-136128, AB_2019997, 1:200), VASP (*65*) (1:1000), WAVE2 (*65*) (1:1000), GFP-nanobody (*66*) (1:1000), glyceraldehyde-3-phosphate dehydrogenase (GAPDH) (Merck Millipore, CB1001-500UG, AB_2107426, 1:1000), α-actinin-1 (this study, 1:1000), FHL3 (this study, 1:1000), α-tubulin (Merck Millipore, MAB1864, AB_2210391, 1:500), nonmuscle myosin 2A (BioLegend, 909801, AB_2565100, 1:500), and pMLC2 (Cell Signaling, 3671S, AB_330248, 1:200). Polyclonal antibodies against tag-free α-actinin-1 and FHL3 were raised by immunizing female New Zealand white rabbits [Charles River, Crl:KBL(NZW)] with respective recombinant proteins following standard procedures. α-Actinin-1 and FHL3 polyclonal antibodies were subsequently purified by affinity chromatography using tag-free antigens conjugated to CNBr-Sepharose 4B (Macherey-Nagel, 17-0430-01).

Primary antibodies in immunoblots were visualized with phosphatase-coupled anti-mouse (Dianova, 115-055-62, 1:1000) or anti-rabbit antibodies (Dianova, 115-055-144, 1:1000). Primary antibodies in immunohistochemistry were visualized with polyclonal Alexa 488–conjugated goat–anti-rabbit (Thermo Fisher Scientific, A-11034, AB_2576217, 1:1000 dilution) or goat–anti-mouse antibodies (Thermo Fisher Scientific, A-11029, AB_2534088, 1:1000), Alexa 555–conjugated goat–anti-mouse antibodies (Thermo Fisher Scientific, A-21424, AB_141780, 1:1000), and Alexa 546–conjugated goat–anti-rat antibodies (Molecular Probes, A-11081, AB_141738, 1:1000 dilution).

### Statement on care and use of animals

The immunization of rabbits for the generation of polyclonal antibodies was conducted in accordance with national guidelines for the care and maintenance of laboratory animals and approved by the Hannover Medical School Institutional Animal Care Facility and the Lower Saxony State Office for Consumer Protection and Food Safety under application number 22-00236 to J.F.

### Immunoblotting

For preparation of total cell lysates, cells were cultured to confluency and trypsinized. Cell pellets were washed twice with ice-cold Dulbecco’s PBS without calcium and magnesium (BioWest, L0615-500) and lysed in ice-cold radioimmunoprecipitation assay buffer (150 mM NaCl, 1.0% Triton X-100, 0.5% sodium deoxycholate, 0.1% SDS, and 50 mM tris, pH 8.0) supplemented with 0.1 mM AEBSF (Carl Roth, 2931.1, 1:1000) and Benzonase (Millipore, E1014, 1:2000) for 30 min at 4°C on a wheel rotor. Subsequently, the lysate was centrifuged at 15,000*g* for 2 min to remove insoluble material. Protein contents of total cell lysates were determined by Pierce BCA assay (Thermo Fisher Scientific, 23225) using a Synergy 4 fluorescence microplate reader (Biotek) according to the manufacturer’s protocol. The clear supernatant was mixed with 3× SDS sample buffer (500 mM tris-HCl, 30% glycerol, 2.9% SDS, and 3% β-mercaptoethanol, pH 6.8) and boiled for 15 min. Total proteins (100 μg) per lane were subjected to SDS–polyacrylamide gel electrophoresis (SDS-PAGE), and transferred by semidry blotting onto nitrocellulose membranes (Hypermol, 6005-01). The membranes were then blocked in NCP buffer (10 mM tris, 150 mM NaCl, 0.05% Tween 20, and 0.02% NaN_3_, pH 8.0) containing 4% bovine serum albumin (BSA) (Sigma-Aldrich, 810531) for 1 hour at room temperature and incubated with primary antibodies in NCP buffer supplemented with 1% BSA overnight. Subsequently, the blots were washed six times for 5 min with NCP buffer and incubated with secondary antibodies conjugated with alkaline phosphatase for 2 to 4 hours. Blots were then washed six times for 5 min with NCP buffer and developed with 5-bromo-4-chloro-3-indolyl phosphate-*p*-toluidine salt (20 mg/ml; Carl Roth, 6368.1) in 0.1 M NaHCO_3_, pH 10.0.

### Immunofluorescence

For immunofluorescence labeling, cells were seeded on FN-coated glass coverslips for 6 hours and then fixed for 20 min in prewarmed PBS, pH 7.4, containing 4% paraformaldehyde (PFA) (AppliChem, 211511) and 0.06% picric acid (Applichem, 251049). Subsequently, the specimens were washed three times with PBS supplemented with 100 mM glycine (Carl Roth, HN07.3), then permeabilized with 0.1% Triton X-100 in PBS for 40 s and blocked for 30 min with PBG (PBS, 0.045% cold fish gelatin and 0.5% BSA). For vinculin stainings, cells were pre-extracted with 0.3% Triton X-100 in 2% PFA in PBS for 2 min and fixed for another 20 min in 4% PFA and 0.06% picric acid in PBS. Primary antibodies diluted in PBG were incubated with the specimens overnight at room temperature, followed by washing three times with PBG. Secondary antibodies diluted in PBG were added for at least 2 hours. The EGFP signal was enhanced with Alexa 488–conjugated nanobodies. To avoid unspecific binding of secondary antibodies to the nanobody, the nanobody was always added after removal of unbound secondary antibody and extensive washing with PBG. Atto550-phalloidin (Atto-Tec, AD550-8, 1:250), Atto488-phalloidin (Atto-Tec, AD 488-8, 1:250), and Atto633-phalloidin (Atto-Tec, AD 633-81, 1:250) were used for visualization of F-actin. DNA was visualized with 4′,6-diamidino-2-phenylindole (Sigma-Aldrich, D8417-1MG, 1:1000). Specimens were mounted in Mowiol (Sigma-Aldrich, 81381). The 12-bit images of fixed cells were captured with an Olympus XI-81 inverted microscope equipped with an UPlan FI 100×/1.30 NA (numerical aperture) oil immersion objective or a Zeiss LSM 980 with Airyscan 2 equipped with a Plan-Apochromat 63×/1.46 NA oil DIC or C-Apochromat 40×/1.20 W Korr objective using the 405-, 488-, 561-, and 633-nm laser lines. Emitted light was detected in the wavelength ranges of 410 to 473, 490 to 545, 576 to 610, and 640 to 693 nm, respectively.

Image analysis was performed using the Fiji image processing software package (*67*). Fluorescence intensities from antibody and phalloidin stainings were measured from images recorded at identical settings to ensure comparability. Background fluorescence intensities were measured outside of the cell. For analysis of FA parameters, confocal images of vinculin-stained cells were subjected to background subtraction using a rolling ball radius of 50 pixels. Subsequently, FAs with a minimum size of 0.25 μm^2^ were segmented and the number of FAs per cell, and their size, length, and mean intensity were derived as parameters using a custom semiautomated ImageJ macro (*19*).

To quantify the decoration of SFs by FHL3, zyxin, and VASP, multichannel confocal stacks of fixed cells stained for respective proteins and additionally counterstained for F-actin with phalloidin were recorded at identical settings and then maximum intensity z-stack projections were generated. To measure unbiased protein intensities along SFs, we selected 10-μm-wide central regions of five SFs per cell in the actin channel, and then determined the mean intensities of the associated proteins as indicated within these defined regions using the “Measure” function in Fiji. SFs containing strain sites were excluded from analysis. Cytoplasmic signals in areas devoid of SFs were then measured accordingly. The background signal was determined outside the cells and then subtracted from both signals before calculating the ratio of the proteins at SFs versus the cytoplasm. The mean intensities of NM2A, pMLC2, and α-actinin-1 at SFs were quantified from cells stained for the respective protein and F-actin as stated above except that the intensities were directly compared after background subtraction. To quantify α-actinin-1 distribution along SFs, central 10-μm-wide SF regions were selected as described above. Peaks in the α-actinin-1 intensity profiles along SFs were detected using the “Find Peaks” function of the BAR plugin in Fiji. The α-actinin-1 peak frequency within the striated pattern of SFs was determined by calculating the number of peaks per micrometer across the line scan. Mean average amplitudes were estimated as the ratio of the maximum and minimum peak values averaged over all peaks of each line scan.

To quantify the fraction of cells with SF strain sites and their average number per cell on substrates of varying stiffness ranging from 0.5 to >10^7^ kPa, control and mDia1/3-KO cells were seeded on an FN-coated Softwell 96 HTS plate (Matrigen, SW96-HTS) for 6 hours. Subsequently, the cells were fixed, permeabilized, and stained for VASP, zyxin, and F-actin. Multichannel confocal stacks were then captured using the LSM 980 equipped with a C-Apochromat 40×/1.20 W Korr objective (Zeiss) and maximum intensity z-stack projections were generated. SF strain sites were counted from randomly arranged images to ensure unbiased analysis.

For quantification of SF width in phalloidin-stained cells, the intensity profiles of line scans perpendicular to single SFs were fitted to the Gaussian function in Fiji. SF width was calculated as the full width at half maximum of the Gaussian. For quantification of SF density, images of phalloidin-stained cells were subjected to a Gaussian blur with a radius of two pixels. Subsequently, straight lines extending beyond the cell edges were drawn to cover the entire SF landscape. Peaks in the intensity profiles along the lines representing SFs were detected using the Find Peaks function of the BAR plugin in Fiji. SF density was determined by calculating the number of peaks per micrometer across the cell, with the outer peaks being considered the cell edge.

### Life-cell imaging and analysis of cell migration and spreading

Time-lapse, phase-contrast imaging of live cells was performed with an Olympus XI-81 inverted microscope (Olympus) driven by Metamorph software (Molecular Devices) and equipped with objectives specified below and a CoolSnap EZ camera (Photometrics). Cells were seeded onto 35-mm glass bottom dishes (Ibidi, 81158), coated for 1 hour with FN (20 μg/ml; Roche) and maintained in growth medium supplemented with 25 mM Hepes, pH 7.0 (Sigma-Aldrich, H3375) to compensate for the lack of CO_2_ in an Ibidi Heating System at 37°C. For 2D random motility assays, the cells were seeded at low density (~5 × 10^4^ cells/ml) onto the dishes and allowed to adhere for 6 hours before imaging. Subsequently, the cells were recorded by time-lapse, phase-contrast imaging at 10-min intervals for 10 hours using an UPlan FL N 4×/0.13 NA objective (Olympus). Single-cell tracking was performed with MTrackJ in ImageJ (*68*). Analyses of cell speed, cell trajectories, and MSDs were performed in Excel (Microsoft) using a customized macro (*69*). Cells that contacted each other or divided were excluded from analysis. Lamellipodial protrusions were recorded at 30-s intervals for 1 hour using an UPlan FL 40×/0.75 NA objective (Olympus). To analyze lamellipodial protrusion at the cell periphery after TCM formation, kymographs were generated from phase-contrast, time-lapse movies in regions with a preexisting lamellipodium or a lamellipodium induced by TCM opening over a period of 11 min using Fiji. Protrusion distances and rates were determined for each frame and correlated with the TCM area over time determined by manual thresholding. For wound healing assays, cells were seeded onto uncoated dishes and expanded to confluency. Subsequently, the monolayer was scratched with a 200-ml pipette tip; the cells were washed three times with warm medium supplemented with 25 mM Hepes, pH 7.0, and recorded by phase-contrast, time-lapse imaging at 10-min intervals for 24 hours using an UPlan FL N 10×/0.30 NA objective (Olympus). Wound closure rates were determined with Fiji software by measuring decrease of scratch area over time.

For 3D migration assays, spheroids were first generated from NIH 3T3 cells and derived mutants. For this purpose, cavities of 96-well microtiter plates (Sarstedt, 83.3924.300) were filled with 40 μl of molten 1% agarose in PBS and allowed to solidify, resulting in a low-adhesive, concave bottom. Then, 2 × 10^3^ cells in 250 μl of growth medium were added to the wells, and the cells were cultivated for another 3 days leading to the formation of compact spheroids. Spheroids were then washed once with ice-cold PBS and embedded in a 20-μl droplet of ice-cold Cultrex R1 extracellular hydrogel matrix (R&D Systems, 3433-005-R1) on the surface of eight-well glass bottom slides (Ibidi). After solidification of the matrix for 30 min at 37°C, the wells were filled with 500 μl of growth medium. Dissemination of cells from spheroids into the hydrogel was captured by phase-contrast imaging at 8- and 16-hour intervals using an Olympus XI-81 inverted microscope equipped with an UPlan FL N 10×/0.30 NA objective. The average migration distance and cell velocity from the surface of the spheroids were derived using Fiji software.

Spreading of wild-type NIH 3T3 cells and derived mutants on FN-coated glass bottom dishes (Ibidi) was monitored at 30-s intervals by time-lapse, phase-contrast imaging for 60 min using an UPlan FL N 10×/0.30 NA objective immediately after seeding. Quantification of cell spreading was performed with Fiji software from time-lapse movies by measuring increase of cell area over time.

For the hypotonic shock experiments, control and mDia1/3-KO cells were seeded on FN-coated glass bottom dishes (Ibidi) and allowed to adhere for 6 hours. The medium was then replaced with 1 ml of growth medium supplemented with 25 mM Hepes, pH 7.0. The cells were imaged at 15-s intervals for 10 min using an Olympus UPlan FL 40×/0.75 NA objective, then 2 ml of sterile double-distilled water was added to reduce the osmolarity from approximately 300 to 100 mosmol, and imaging continued for an additional 30 min.

### TIRF life-cell imaging

NIH 3T3 fibroblasts and derived mutants transfected for 16 to 24 hours were plated on FN-coated 35-mm glass bottom dishes (Ibidi). After 6 hours, the medium was supplemented with 25 mM Hepes (Sigma, H0887). Imaging was carried out using a Nikon Eclipse TI-E inverted microscope equipped with an Ibidi stage-top heating system at 37°C. For analysis of FA dynamics, cells transfected with pEGFPN1-paxillin were imaged at 1-min intervals for 10 hours using a TIRF Apo 100× objective in combination with a 488-nm laser. For visualization of TCM opening and closure, cells were transfected with pmScarlet-LifeAct and pEGFPC1-CAAX for detection of endogenous F-actin or pEGFPC1-α-actinin-1 at 30-s intervals for appropriate time periods using a TIRF-Apo 100× objective in combination with the 488-nm and 546-nm laser lines, respectively.

For quantification of TCM closure, EGFP-α-actinin-1 intensities were visualized over time using the “3D Surface Plot” function in Fiji. TCM closure was analyzed by tracking the mean interpeak distance of four consecutive EGFP-α-actinin-1 peaks at the TCM edge. For this purpose, a four-pixel-thick line was drawn over the region to be analyzed, and its position was adjusted over time as the TCM edge moved as it closed. Peaks in the EGFP-α-actinin-1 intensity profiles across the line over time were detected using the Find Peaks function of the BAR plugin in Fiji. Mean interpeak distances were calculated within the area delimited by the outer peaks.

### Analysis of FA dynamics

FA dynamics was analyzed by TIRF imaging of cells expressing low levels of EGFP-paxillin randomly migrating on FN-coated 35-mm glass bottom dishes (Ibidi) using the TIRF setup described above. Autocorrelation analysis was performed as previously described (*70*). Briefly, a mask was created by manually thresholding the first frame of a 30-min movie to define the area of the FAs. The sum of the intensities of all pixels within the mask for all frames was normalized to the sum of the intensities in the first frame. This measure of autocorrelation defines turnover as the change in area, location, and intensity of adhesions, and not as the rate at which molecules are exchanged between FAs and the cytosol. However, all of these processes are indicative of disassembly, and we consider this autocorrelation index to be an indicator of FAs dynamics where an autocorrelation value of one indicates no change and a value of zero indicates complete loss of structure.

For the analysis of FA lifetime and sliding velocity, individual paxillin plaques that appeared and disappeared during the observation period were manually tracked throughout their lifetime using the MTrackJ plugin in ImageJ and categorized as FAs originating from lamellipodia or the cell body. FAs that split or merged with other FAs were excluded from the analysis. To calculate the fraction of FAs that split or merged with other FAs during their lifetime, all FAs present at a given time were tracked until they disappeared, fused, or split, and categorized accordingly as stable, splitting, or merging FAs. In each case, the first event was counted and the adhesion was not tracked thereafter. Assembly and disassembly rates were inferred as previously described (*34*) with modifications using Fiji software. Briefly, regions of interest (ROIs) were manually superimposed on individual paxillin plaques that appeared and disappeared during the imaging period. ROIs have been adjusted over time where necessary. The background ROIs were derived by shifting the original time-encoded ROIs to nearby locations within the cells that were devoid of FAs. Fluorescence intensity values were measured over time and corrected for background intensities. Disassembly rate constants *k*_dis_ were derived by fitting the fluorescence intensities [*I*_disassembly_(*t*)] over time *t* of the decay phase to

$$I_{\text{disassembly}}\left(t\right)=f_{0}e^{-k_{\text{dis}}\left(t-a\right)}$$

using the Solver add-on in Microsoft Excel by minimizing the sum of squares of the residuals with *f*_0_, *a*, and *k*_dis_ as variables. Here, *a* is defined as the offset of the exponential function at the ordinate and *f*_0_ is defined as the intensity at *t* = *a*. The assembly rate constant *k*_ass_ was calculated by fitting the function

Iassembly(t)=fmax1+e−kass(t−t12)

to the ascending and steady phase of the intensity distribution over time. The maximum fluorescence intensity *f*_max_, the time at half-maximum *t*_1/2_, and the rate constant *k*_ass_ were set as variables.

### Micropipette aspiration assay

Micropipette aspiration was carried out as previously described (*18*). The setup was described in detail previously (*71*). Briefly, a chamber with one open side was filled with 1 ml of PBS buffer and mounted on a stage of an inverted Axiovert 200 microscope (Zeiss) equipped with an LD Achroplan 40×/0.6 objective (Zeiss). A BSA-coated glass micropipette with an inner diameter of 4.6 ± 1.3 μm was also filled with PBS buffer and positioned into the measurement chamber using a micromanipulator (Narishige). Aspiration pressure was applied with a height-adjustable water reservoir. The reference pressure (0 Pa) was calibrated by observing the motion of 2-μm polystyrene beads (Polysciences, 19814-15) in the pipette. After setting the pressure difference to 50 Pa, 50 μl of cell suspension was carefully injected into the chamber, and only nonadhering cells were aspirated. Aspiration was recorded using a sensicam qe CCD (charge-coupled device) camera (PCO) with a frame rate of at least 2 Hz. Aspiration length (*L*_p_) was determined using OpenBox (Informationssysteme Schilling) and Fiji software.

### Single-cell force spectroscopy

Before experiments, glass-bottomed dishes were functionalized to allow analysis of highly and weakly adhering cells. For strong adhesion, the surface was coated with 0.5% FN for 1 hour at 37°C after oxygen plasma activation for 30 s. To achieve strongly reduced adhesion, the surface was uniformly coated with a polyethylene glycol (PEG) gel (*72*). To this end, the glass was first washed with water and ethanol, followed by oxygen plasma cleaning for 10 s, and then exposed to a vapor of 3-(Trimethoxysilyl) propyl methacrylate (Sigma-Aldrich, 440159) at 77°C for 30 min. A 30% polyethylene glycol diacrylate (PEGDA) (Sigma-Aldrich, 455008) prepolymer solution was then added to the center of the dish containing the UV-sensitive photo-initiator VA-086 (Fujifilm Wako Chemicals Europe, 61551-69-7), which cross-links PEG-DA molecules via acrylate groups and converts the PEGDA to a PEG gel by exposure to 2.19 J cm^−2^ of 365 nm UV radiation for 60 s, resulting in a 4-μm-thick layer.

Single-cell force spectroscopy (SCFS) was carried out using a vibration isolated MFP-3D AFM (Asylum Research) with an extended z-scanner (40 μm nominal range) mounted on a bright-field IX71 Olympus microscope (Shinjuku City, Tokyo, Japan) with a 40× (LUCPlanFLN, NA = 0.6) Olympus objective connected to a Zyla sCMOS camera (Andor). Force measurements were performed with untreated ArrowTM TL2 tipless cantilevers [NanoWorld; Neuchatel, Switzerland; nominal spring constant (*k*_c_) = 0.03 N m^−1^] in contact to single cells prepared in DMEM and maintained at 37°C within a petri Dish Heater (Asylum Research). Spring constant calibration followed the thermal noise method (*73*). Experiments started 30 to 60 min after sedimentation to the functionalized substrates mentioned above by obtaining compression-relaxation curves after assessing the uncompressed cell radius. Because the tipless cantilever is considerably larger compared to the cells, the entire surface area was measured. Force curves were obtained at a speed of 1 μm s^−1^, a contact force of 1 to 2 nN, and a dwell time of 5 s. The indentation depth was kept below 50 to 250 nm to mainly probe the actin cortex of the cells and the yield force was set to 0.5 nN as a compromise between dilating the area and avoiding touching the nucleus. Force compression-relaxation curves were analyzed following the viscoelastic Evans model, as previously described (*74*), using a custom-written Python/Matlab routine requiring cell radius, baseline drift removal, hydrodynamic drift correction, and contact point selection. The routine calculated the geometry of the cell under compression for determination of the prestress *T*_0_ (cortical tension) in dependence of the spreading state and indenter geometry.

### STED imaging of fixed and live cells

NIH 3T3 fibroblast and derived mutant cells were seeded in growth medium on high-precision glass cover slips with a thickness of 0.17 mm (Marienfeld Superior, 0117580) coated with FN (20 μg/ml; Roche, 11051407001) in six-well plates (Sarstedt, 83.3920300). After 6 hours, the cells were briefly washed with prewarmed (37°C) PBS, and then fixed and permeabilized with 0.3% glutaraldehyde and 0.2% Triton X-100 (AppliChem, A1388,0500) in cytoskeleton buffer (10 mM MES, pH 6.1, 150 mM NaCl, 5 mM EGTA, 5 mM glucose, and 5 mM MgCl_2_) for 2 min. Subsequently, the cells were postfixed with 2% glutaraldehyde (Carl Roth, 4157.2) in cytoskeleton buffer for 10 min. The reduction was carried out with 0.1% sodium borohydride (Sigma-Aldrich, 213462-25G) for 5 min. The cells were then washed five times with PBS, and stained with STAR635-conjugated phalloidin (Abberior, ST635-0100-20UG) at room temperature overnight to visualize F-actin. After extensive washing with PBS, the samples were mounted in Mowiol supplemented with 0.1% (v/v) 1,4-Diazabicyclo[2.2.2]octan (DABCO, 8.03456). STED nanoscopy with fixed cells was performed using an Expert Line dual-color STED 775 QUAD scanning microscope (Abberior Instruments) equipped with an UPlanSApo 100×/1.40 oil [infinity]/0.17/FN26.5 objective (Olympus). The fluorophore STAR635 was excited at 640 nm wavelength and STED was performed at 775 nm wavelength. The fluorescence emissions were recorded with an avalanche photodiode using time-gated detection (0.75 to 8 ns) in the spectral window of 650 to 720 nm. The microscope was controlled by the Imspector software (Abberior Instruments; version 16.1.7.128). Images were recorded at a pixel size of 18 to 20 nm in the 2D STED mode. The pinhole was set to 0.6 to 0.7 AU. To quantify phalloidin intensities at the cell cortex, central regions distant from the nucleus and the cell periphery, which also lack SFs and thus mainly represent the cortical F-actin cytoskeleton, were randomly selected from images recorded at identical settings. In selected regions, five 10-μm-long lines with a thickness of five pixels were defined and intensity values were obtained by using the “Measure” function in Fiji. The background signal was determined outside the cells and then subtracted from the measured values. Quantification of phalloidin intensities at SFs was performed accordingly, except that lines were placed in the central regions of randomly selected SFs.

For STED imaging of live cells, cells were seeded in eight-well chamber slides (Ibidi, 80821) coated with FN (20 μg/ml) 16 to 18 hours before imaging. The cells were stained with DMEM containing 0.4% (v/v) SPY650-FastAct (Spirochrome, SC505) for 1 hour at 37°C and were recorded with the same imaging setup described above without washing. SPY650-FastAct was excited at 640 nm wavelength, and stimulated emission depletion was performed at 775 nm wavelength. The fluorescence emissions were recorded with an avalanche photodiode using time-gated detection (0.55 to 8 ns) in the spectral window of 650 to 720 nm. Images were recorded at a pixel size of 40 nm in the 2D STED mode. The pinhole was set to 0.7 AU. The pixel dwell time was set to 10 μs. Each line was scanned once in the confocal mode and seven times in the STED mode.

### Scanning electron microscopy

For scanning EM experiments, glass coverslips (Scientific labs UK, 12 mm diameter, no. 1.5) were washed with detergent and stored in 70% ethanol. The coverslips were then removed from ethanol, dried in the hood on tissue paper, placed on the bottom of Nunc 4-well plates (Thermo Fisher Scientific, 179830), and coated with 50 μl of FN at a final concentration of 20 μg/ml for 30 min. Subsequently, 50 μl of cell suspension with a density of 1 × 10^5^ cells/ml in growth medium was added to the FN-coated coverslips and allowed to attach for 30 min, before flooding the well with growth medium. Six hours later, the cells were washed in PBS, before being permeabilized for 7 min on ice in prechilled PEM buffer (80 mM Pipes; pH 6.9, 2 mM MgCl_2_ and 0.5 mM EGTA) supplemented with 0.01% saponin (Sigma-Aldrich, 47036), 2 μM phalloidin (Sigma-Aldrich, P2141), 10 μM taxol (Sigma-Aldrich, PHL89806), and 300 mM sucrose (Sigma-Aldrich, S0389). The coverslips were then removed, briefly dipped into PEM buffer supplemented with 1% Triton X-100 (Thermo Fisher, 85111), and the cells were subsequently fixed with 2% glutaraldehyde (Sigma-Aldrich, G5882) in PEM buffer supplemented with phalloidin (10 μg/ml) for 5 min at room temperature and then overnight at 4°C. The following day, the cells were postfixed with 0.1% tannic acid (Agar Scientific, Y2863) in water for 20 min, washed five times with double-distilled H_2_O, and postfixed in 0.2% uranyl acetate (BDH Chemicals, 10288) in double-distilled H_2_O. The cells were then critically point dried (*18*). The coverslips were mounted onto SEM stubs and coated with carbon to a thickness of less than 10 nm using a Quorum unit. Specimens were imaged using a Hitachi SUX230 high-resolution FEG SEM at a range of magnifications from 3 to 5 K, to 50 K. Regions distant from the nucleus and the cell periphery were randomly chosen from the SEM images to measure pore size within the cortical actin meshwork. For each selected region, the average diameter of 40 randomly chosen pores was measured manually using the “Straight Line” and “Measure” tools in Fiji.

### Cell stretching experiments

Elastic silicone substrates for stretch experiments were prepared and calibrated as previously described (*75*). Cell straining experiments were performed on 50-kPa elastomeric stretching chambers (Dow Corning, Sylgard 184, mixing ratio of 1 to 40 cross-linker to base) to mimic natural elasticities. Elasticities were determined by indentation as previously described (*76*). To promote cell attachment, chambers were coated with human FN (20 μg/ml) (Corning, 354008) in PBS at 37°C for 1 hour before cell seeding. Cells were seeded for 18 hours before cell straining at a density of 1 × 10^4^ cells/cm^2^. Uniaxial stretch was applied to six elastic substrates simultaneously as previously described (*77*). Cells were stretched for 1 hour with 300 mHz and 20%, and then fixed in stretched state with 4% PFA in PBS. After permeabilization with 0.1% Triton X-100 for 45 s, filamentous actin was stained with Alexa Fluor-488 phalloidin (Life Technologies, A12379). Images were captured with a Zeiss AxioObserver wide-field microscope equipped with a 40× EC-PlanNeofluar/Ph3/1.3 NA oil immersion objective.

### Traction force microscopy

For cell force analysis, cells were seeded on FN-coated 15-kPa elastomeric Sylgard 184 silicone rubber substrate (Sigma-Aldrich, 761036). To visualize and analyze the deformation of the elastic substrate, red fluorescent beads (0.2-μm FluoSpheres Crimson carboxylate-modified beads, Invitrogen, F8806) were covalently coupled as previously described (*78*). Live cell analysis was performed at 37°C and 5% CO_2_ using an AxioObserver wide-field microscope (Zeiss). Details on experimental procedures and force calculation have been previously described (*79*, *80*).

### Fluorescence lifetime imaging microscopy

For live-cell FLIM analysis, cells were seeded on FN-coated glass bottom imaging dishes (Ibidi, 81158) and allowed to adhere and form FAs. Living cells were imaged in phenol-red free DMEM supplemented with 10% fetal calf serum. FLIM experiments were performed with a TCS SP5 X confocal laser scanning microscope (Leica) equipped with a pulsed white light laser (80 MHz repetition rate, NKT Photonics), a band-pass filter for YPet (545/30 nm, Chroma), a FLIM X16 TCSPC (time-correlated single photon counting) detector (LaVision Biotech), a 63× water objective (HCX PL APO CS, NA = 1.2), and a heating chamber (37°C, 5% CO_2_; Ibidi). The analysis of the resulting TCSPC-FLIM data was performed using custom-written MATLAB programs (*81*, *82*). In brief, a multi-Otsu thresholding algorithm was applied to isolate FA specific signals using the donor intensity image. The fluorescence lifetime was determined by fitting an exponential decay to the photon count histogram of each masked cell. To minimize the contribution of the instrument response function and auto-fluorescence, fitting was started 0.56 ns after the maximum photon count. The FRET efficiency *E* was calculated from the lifetime of the donor in presence of an acceptor τ_DA_ and the average donor-only lifetime $\bar{\tau_{D}}$, according to

$$E=1-\frac{\tau_{\text{DA}}}{\;\bar{\tau_{D}}}$$

### Mass spectrometry

MS-based proteome analyses were done as described recently (*83*). Briefly, proteins were mixed and alkylated by acrylamide, and further processed by SDS-PAGE and Coomassie Brilliant Blue (Sigma, 1154440025) staining. After destaining and extensive rinsing with bi-distilled water, two gel pieces in the range of 35 to 55 and 70 to 200 kDa, respectively, were excised from each lane to allow for identification of Arp2/3 complex components and all possible formins, and then in-gel digested with sequencing grade Trypsin (Promega, V5111). Peptide samples were analyzed with data-dependent analysis in a liquid chromatography–mass spectrometry (LC-MS) system (RSLC, Orbitrap Exploris 240, both Thermo Fisher Scientific). Raw MS data were processed using Max Quant software (version 2.5) and Perseus software (version 2.0.6.0) and mouse entries of UniProt database. Proteins were stated identified by a false discovery rate of 0.01 on protein and peptide levels. As possible modifications, oxidation at methionine, deamidation at asparagine and glutamine, and N-terminal acetylation were considered. Protein intensity values were normalized for each sample by subtracting median values of each sample to correct different protein loads. The relative abundance of identified actin nucleators was subsequently normalized to the intensities of the 50 proteins that exhibited the smallest deviations between wild-type and mutant cells.

### Statistics and reproducibility

Quantitative experiments were performed at least in triplicates to avoid any potential environmental bias or unintentional error. Specific phenotypes as derived from analyses on fixed samples or living cells were systematically obtained from sample sizes of dozens or hundreds of cells, respectively. Raw data were processed in Excel (Microsoft). No samples or data points were omitted from analysis. Statistical analyses were performed with Origin 2021 (OriginLab). All datasets were tested for normality by the Shapiro-Wilk test. Statistical differences between normally distributed datasets of two groups were determined by *t* test and not normally distributed datasets of two groups by nonparametric Mann-Whitney *U* rank sum test. For comparison of more than two groups, statistical significance of normally distributed data was examined by one-way analysis of variance (ANOVA) and Tukey’s multiple comparison test. In case of non-normally distributed data, the nonparametric Kruskal-Wallis test and Dunn’s multiple comparison test were used. Statistical differences were defined as *P < 0.05, **P < 0.01, ***P < 0.001, and not significant (n.s.). All values are shown as means ± SD or means ± SEM. All data values for experiment with small sample sizes are provided in table S2.
