# Supplementary material for: Decisive role of mDia-family formins in cell cortex function of highly adherent cells
Source: Sci Adv. 2024 Oct 30;10(44):eadp5929. doi: 10.1126/sciadv.adp5929 (PMC11524191; doi:10.1126/sciadv.adp5929)
Supplement: Supplementary file 1 — Figs. S1 to S14 Table S1 Legend for table S2 Legends for movies S1 to S11 [file sciadv.adp5929_sm.pdf]

Science Advances
AAAS

Supplementary Materials for

Decisive role of mDia-family formins in cell cortex function of highly adherent cells

Jonas Scholz *et al.*

Corresponding author: Jan Faix, faix.jan@mh-hannover.de

Sci. Adv. **10**, eadp5929 (2024)
DOI: 10.1126/sciadv.adp5929

The PDF file includes:

Figs. S1 to S14
Table S1
Legend for table S2
Legends for movies S1 to S11

Other Supplementary Material for this manuscript includes the following:

Table S2
Movies S1 to S11

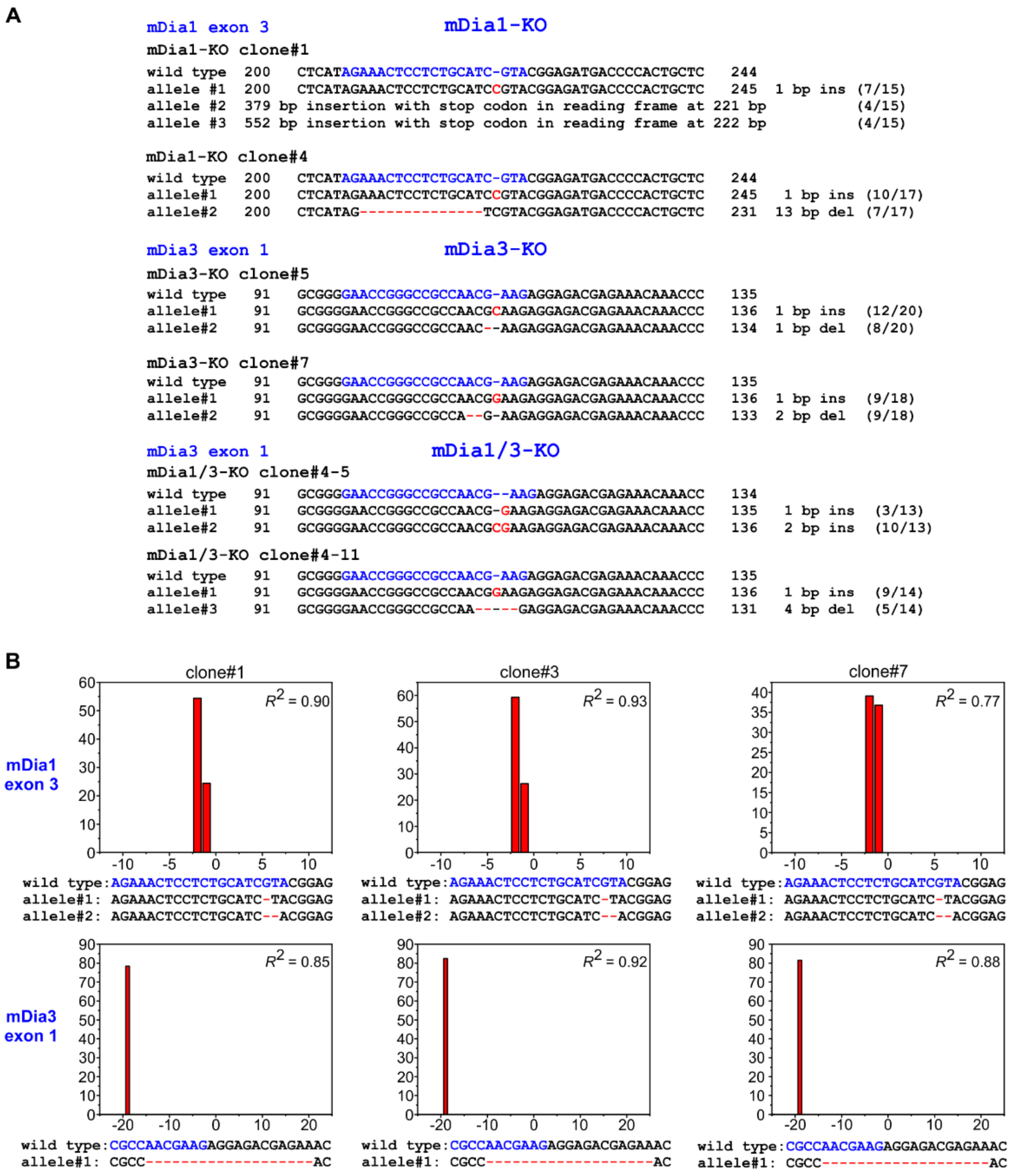

Fig. S1. Confirmation of lack of mDia1 and mDia3 expression in respective KO lines. (**A**) Alignments of genomic target sequences from CRISPR/CAS9-generated NIH 3T3 mDia-mutants with respective wild-type alleles. DNA sequence alignments of wild-type mDia1 exon-3 and mDia3 exon-1 fragments *versus* all alleles identified in mDia-KO mutant cells lines by DNA sequencing, including respective mutations. Blue letters in respective wild-type sequences denote CRISPR/Cas9 target sites. Red letter or dashed lines indicate either frame-shift insertions or deletions, respectively. Numbers in brackets following each cell clone indicate the frequency of occurrence in respective alleles. (**B**) The mDia1/3-KO double mutants (clones #1, #3 and #7), derived from CF-1 fibroblasts, were identified by the TIDE sequence trace decomposition web tool. R^2 indicates goodness of fit.

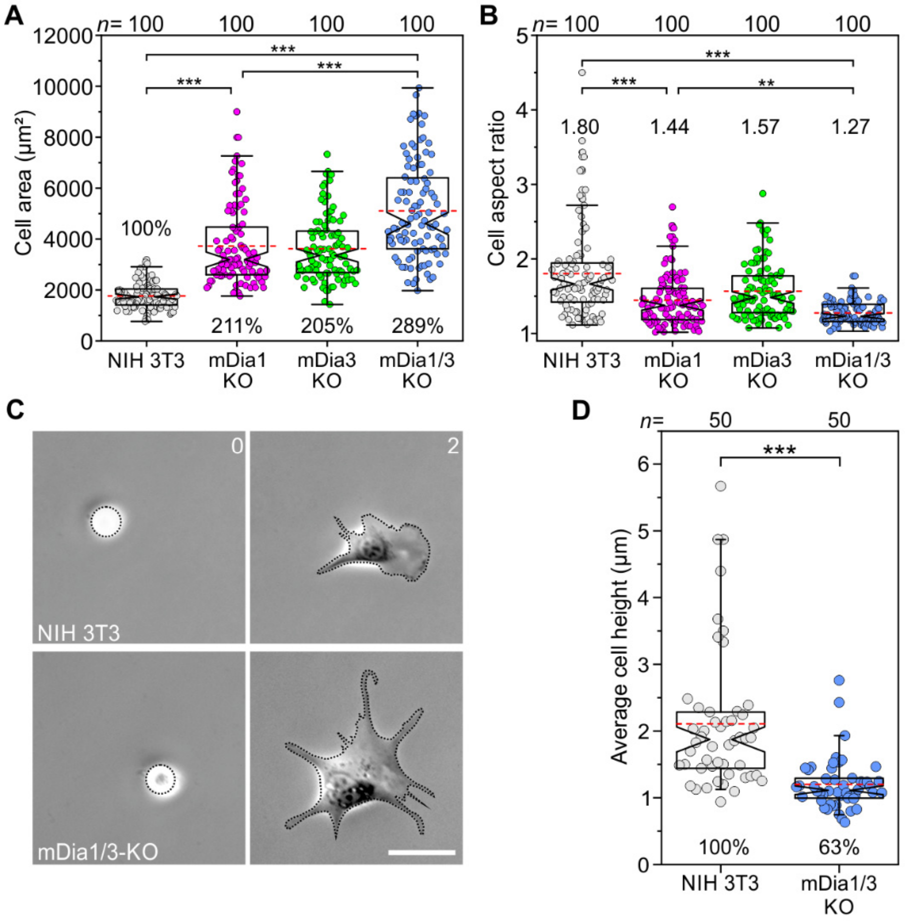

Fig. S2. Loss of mDia1/3 increases cell area and diminishes cell polarity and height. (**A**) Quantification of cell area in control and derived mutants as indicated. (**B**) Quantification of cell aspect ratio as a measure of polarity. (**C**) Representative phase-contrast images of control and mDia1/3-KO cells prior and after spreading on FN. Time is indicated in h. Scale bar, 50 µm. (**D**) Quantification of cell height assessed by calculating the ratio of the cell volume of freshly trypsinized, non-adherent cells to the cell area 2 h after seeding cells on FN-coated glass bottom dishes. (A,B,D) Boxes in box plots indicate 50% (25-75%) and whiskers 90% (5-95%) of all measurements, with dashed red lines depicting the means. Medians are highlighted by indentation of boxes. Results are pooled data from five biologically independent replicates. *n*, number of cells analyzed. Percentages and mean values and are shown to better illustrate the differences between cell lines. (A,B) Kruskal-Wallis test with Dunn's multiple comparison test. **P < 0.01 and ***P < 0.001. (D) Mann-Whitney-U-test test. ****P* < 0.001.

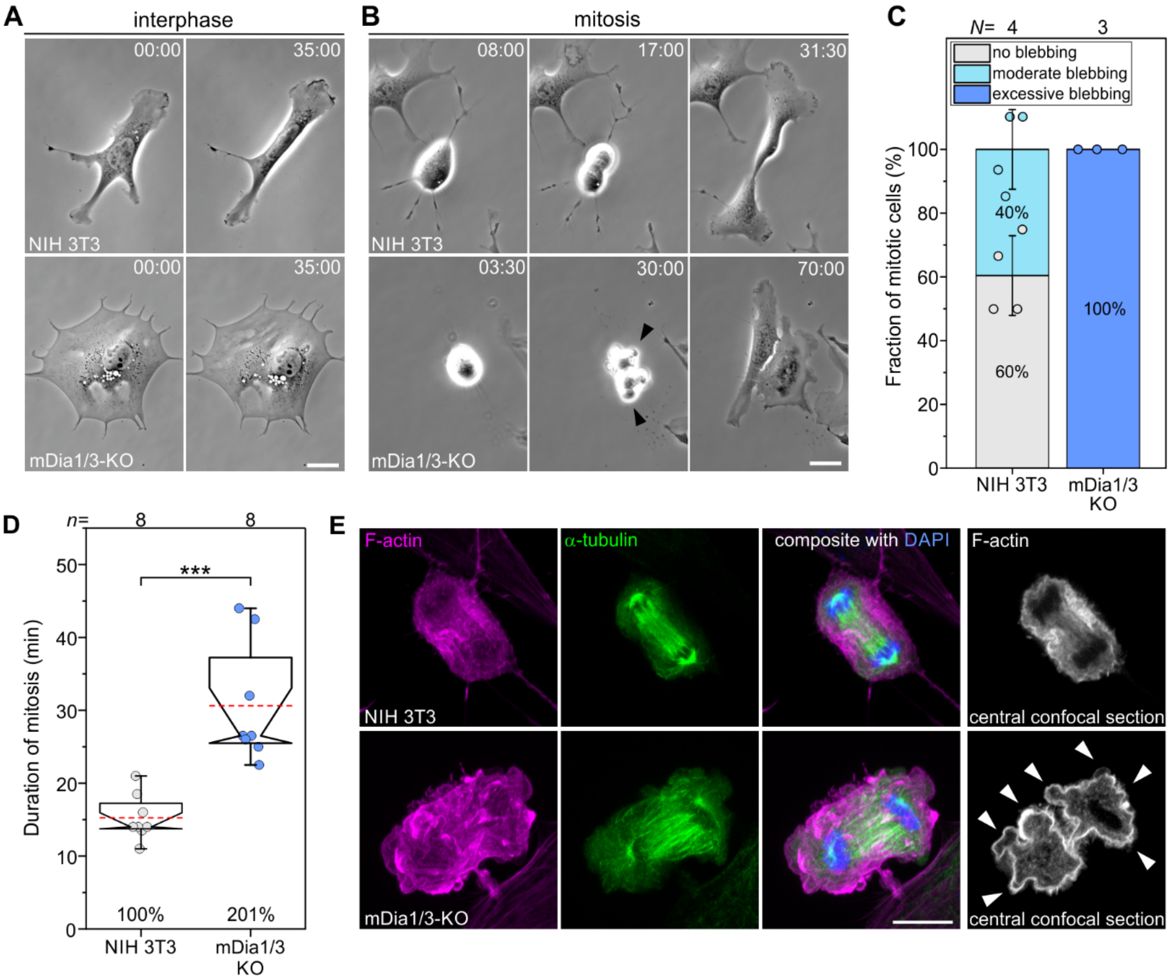

Fig. S3. mDia1/3-KO cells exhibit excessive blebbing cells during mitosis. (**A** and **B**) Representative images from time-lapse, phase-contrast movies of control NIH 3T3 and mDia1/3-KO cells on FN during the interphase and mitotic cell division; data correspond to movie S2. Scale bars, 20 µm. Time is indicated in min and sec. Note excessive blebbing of the mDia1/3-KO mutant during mitosis, which is not seen in adherent cells during interphase. (**C**) Quantification of blebbing in control and mutant cells. *N*, number of biological replicates. Data indicate mean ± SD. Data points indicate means of individual experiments with at least 3 cells each. Percentages are shown to better illustrate the differences between cell lines. (**D**) Quantification of the duration of mitosis in control and mDia1/3-KO cells. Boxes in box plots indicate 50% (25-75%) and whiskers 90% (5-95%) of all measurements, with dashed red lines depicting the means. Medians are highlighted by indentation of boxes. Mann-Whitney-U-test test. Results are pooled data from at least 3 biologically independent replicates. Percentages are shown to better illustrate the differences between cell lines. *n*, number of cells analyzed. (**E**) Representative confocal z-stack projections of dividing control and mDia1/3-KO cells stained for the F-actin cytoskeleton with phalloidin, α-tubulin and DNA with Dapi. Scale bar, 5 µm. A central confocal section is shown on the right to better illustrate the presence of multiple blebs (white arrow heads) in the double mutant. ***$P < 0.001$.

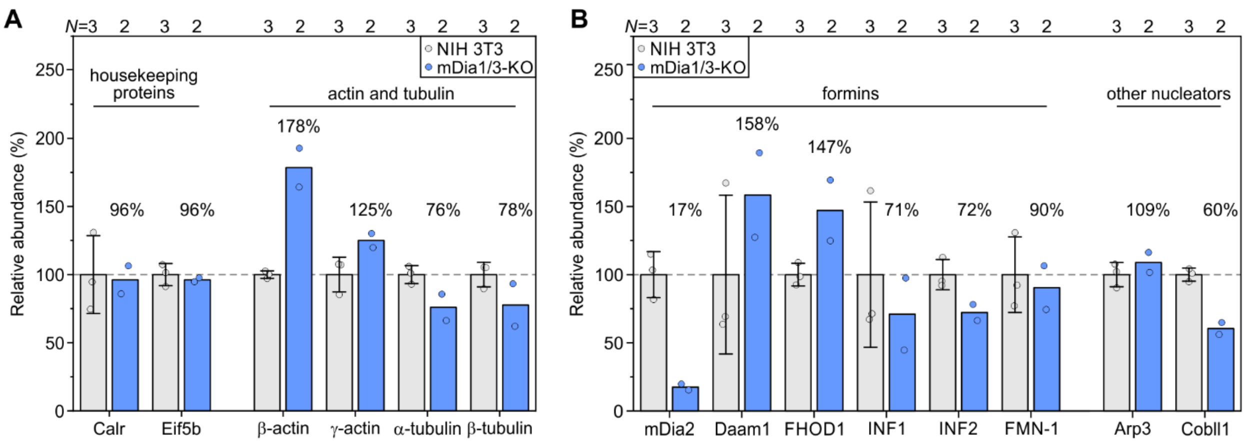

Fig. S4. Combined loss of mDia1/3 leads to changes in the abundance of other actin nucleators. (A) Quantification of relative protein levels of the housekeeping proteins calreticulin (Calr) and eukaryotic translation initiation factor 5B (Eif5b) as well as of actin and tubulin isoforms by mass spectrometry in control and mDia1/3-KO cells. Notably, both actin isoforms appear to be upregulated in mDia1/3-KO mutant cells, whereas tubulin isoforms were slightly downregulated. (**B**) Quantification of protein abundance of formins and other actin nucleators by mass spectrometry. (A,B) Expression levels of proteins were normalized to protein load and the 50 proteins exhibiting the smallest deviations between wild-type and mutant cells. Bars and error bars represent means ± SD. Data points indicate individual experiments. *N*, number of biological replicates. Percentages are shown to better illustrate the differences between cell lines. Of note, FMNL3 was not identified in any of the three replicates of control cells but was clearly found in both replicates of the double mutant, suggesting an upregulation as seen for Daam1 and FHOD1 upon combined loss of mDia1/3. The remaining members of the formin family were either below the detection limit or not expressed in both cell lines.

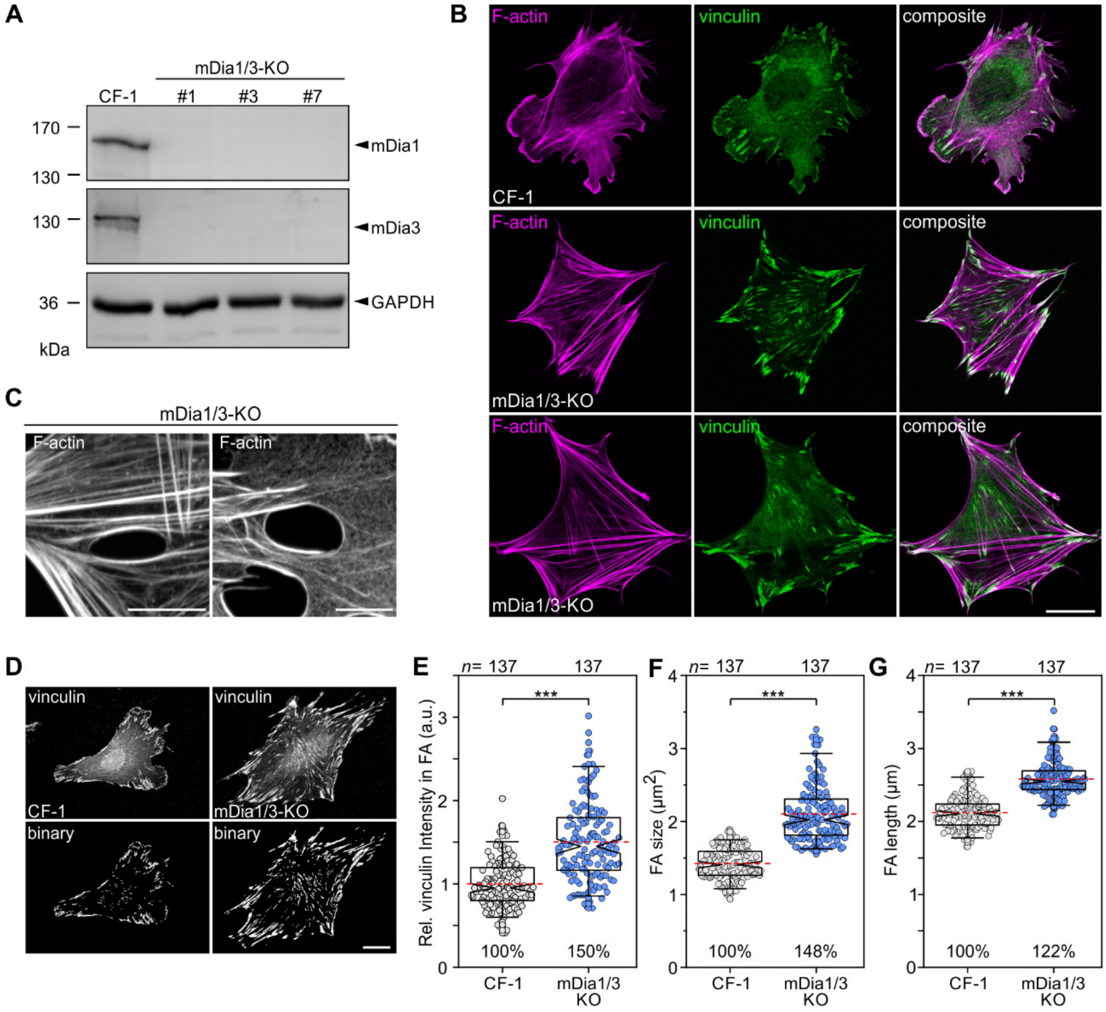

Fig. S5. Loss of mDia1/3 in CF-1 fibroblasts amplifies FA formation and induces the formation of macroapertures. (**A**) Combined loss of mDia1 and 3 in CF-1 fibroblasts in independent mutants was confirmed by immunoblotting. GAPDH was used as loading control. (**B**) Representative confocal z-stack projections of control CF-1 and independent double-mutant cells seeded on FN and stained for vinculin and the F-actin cytoskeleton. Scale bar, 20 µm. Note stretched morphology and formation of prominent SFs and FAs in the double mutant cells as opposed to control. (**C**) Representative confocal z-stack projections of cellular macroapertures formed by CF-1 mDia1/3-KO cells. Scale bars, 10 µm. (**D**) Representative confocal z-stack projections of a CF-1 control and mDia1/3-KO CF-1 cell displaying vinculin staining before (upper panel) and after binarization (lower panel). Scale bar, 20 µm. (**E**) Quantification of vinculin intensities in FA. (**F**) Quantification of FA size. (**G**) Quantification of FA length. (E-G) Boxes in box plots indicate 50% (25-75%) and whiskers 90% (5-95%) of all measurements, with dashed red lines depicting the means. Medians are highlighted by indentation of boxes. Mann-Whitney-U-test test. Results are pooled data from 5 biologically independent replicates. Percentages are shown to better illustrate the differences between cell lines. *n*, number of cells analyzed. ***$P < 0.001$.

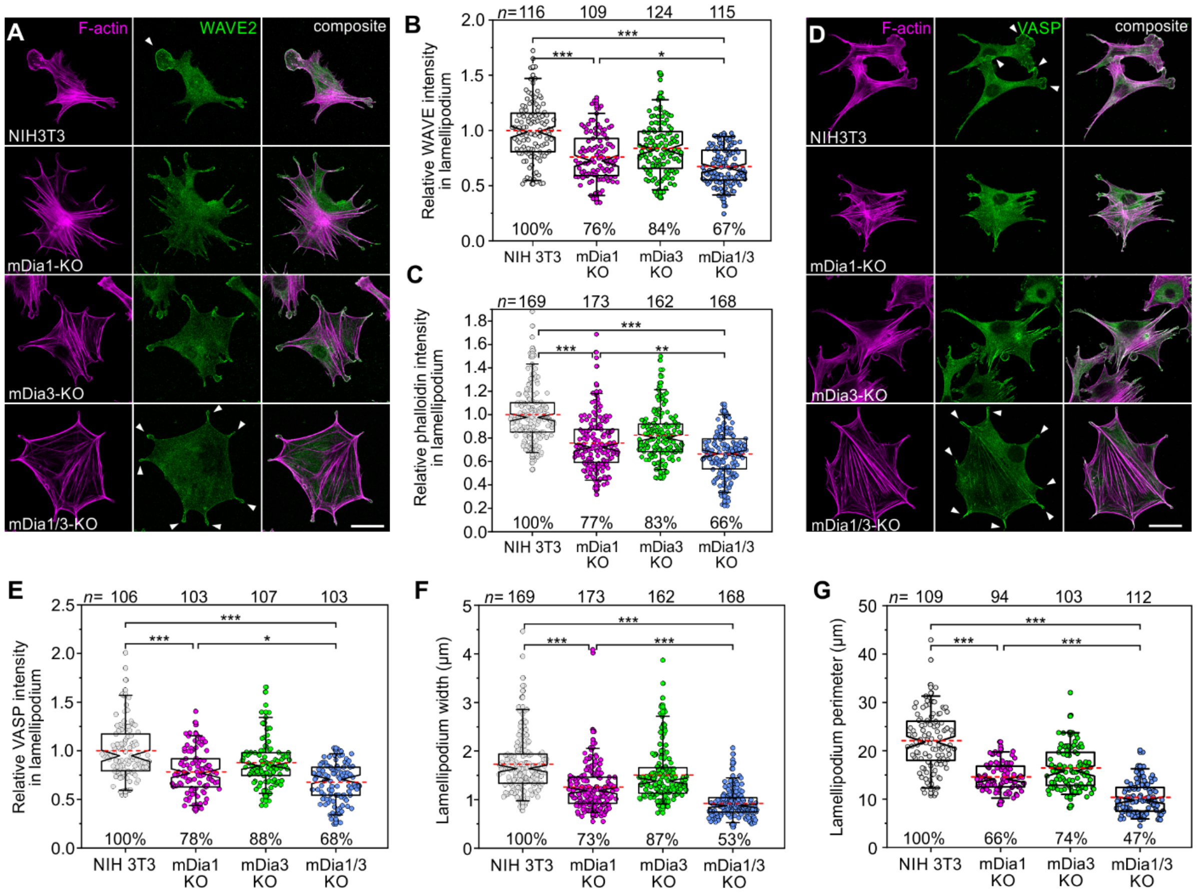

Fig. S6. Individual and combined loss of mDia1 and mDia3 impairs lamellipodium formation. (**A**) Representative confocal z-stack projections of control and mutant cells seeded on FN and stained for WAVE2 and the F-actin cytoskeleton. Fronts are indicated by white arrowheads. Scale bar, 20 µm. (**B**) Quantification of WAVE2 intensity in lamellipodia. (**C**) Quantification of phalloidin intensity in lamellipodia. (**D**) Representative confocal z-stack projections of control NIH 3T3 and mutant cells seeded on FN and stained for VASP and F-actin. Fronts are indicated by white arrowheads. Scale bar, 20 µm. (**E**) Quantification of VASP intensity in lamellipodia. (**F**) Quantification of lamellipodium width. (**G**) Quantification of lamellipodium perimeter. (B-C, E-G) Boxes in box plots indicate 50% (25-75%) and whiskers 90% (5-95%) of all measurements, with dashed red lines depicting the means. Medians are highlighted by indentation of boxes. Kruskal-Wallis test with Dunn's multiple comparison test. Results are pooled data from at least 3 biologically independent replicates. Percentages are shown to better illustrate the differences between cell lines. *n*, number of cells analyzed. $^{*}P < 0.05$, $^{**}P < 0.01$ and $^{***}P < 0.001$.

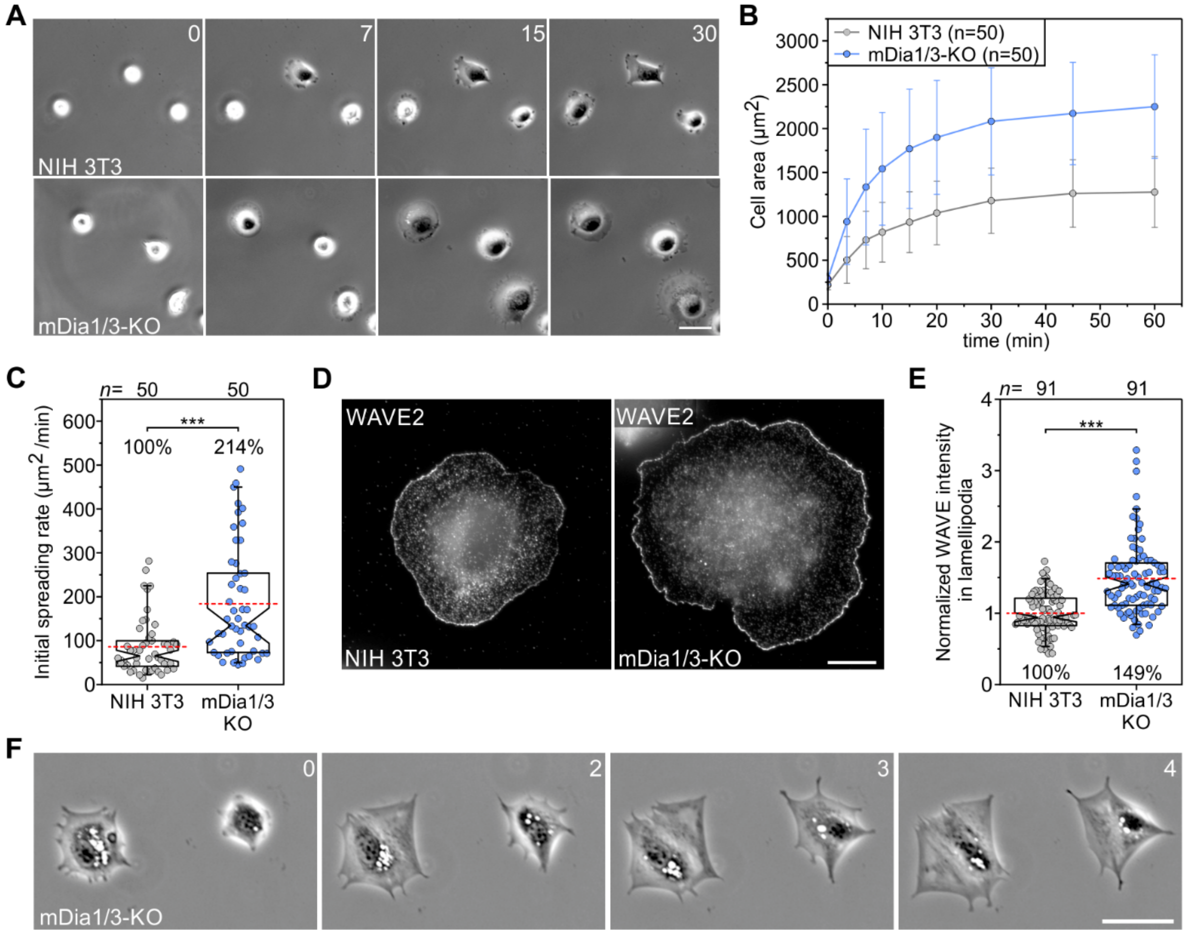

Fig. S7. Combined loss of mDia1 and mDia3 promotes cell spreading. (**A**) Spreading of control and mDia1/3-KO cells on FN. Time is indicated in min. Scale bar, 50 µm. (**B**) Quantification of cell area over time. Data indicate mean ± SD. *n*, number of analyzed cells from five (control) or nine (mDia1/3-KO) independent movies. (**C**) Quantification of initial spreading rate. *n*, number of analyzed cells from five (NIH 3T3) or nine (mDia1/3-KO) independent movies. (**D**) Representative epifluorescence images of fixed cell during initial phase of spreading stained for the protrusion marker WAVE2. (**E**) Quantification of WAVE2 intensity in lamellipodia of spreading cells. *n*, number of cells analyzed from 3 biologically independent replicates. (**F**) Development of the mDia1/3-KO specific phenotype on FN over time. Representative stills of time lapse, phase-contrast movies are shown; data correspond to movie S3. Time is indicated in h. Scale bar, 50 µm. (C,E) Boxes in box plots indicate 50% (25-75%) and whiskers 90% (5-95%) of all measurements, with dashed red lines depicting the means. Medians are highlighted by indentation of boxes. Mann-Whitney-U-test test. Results are pooled data from at least 3 biologically independent replicates. Percentages are shown to better illustrate the differences between cell lines. ***$P < 0.001$.

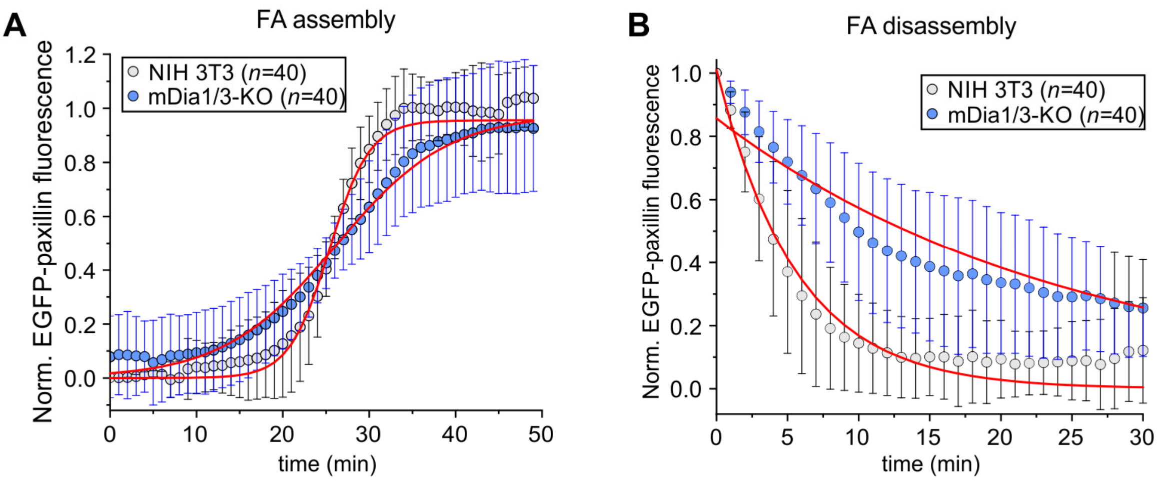

Fig. S8. Combined loss of mDia1/3 in NIH 3T3 fibroblasts alters FA dynamics. (**A**) Quantification of FA assembly in control cells and mDia1/3-KO mutants expressing EGFP-paxillin on FN over time. Red lines denote logistic fits. (**B**) Quantification of FA disassembly in control NIH 3T3 cells mDia1/3-KO mutants expressing EFGP-paxillin on FN over time. Red lines denote exponential fits. Data correspond to Fig. 4D and E. (A,B) Data points and error bars represent means ± SD from 5 biologically independent replicates each. *n*, number of FAs analyzed.

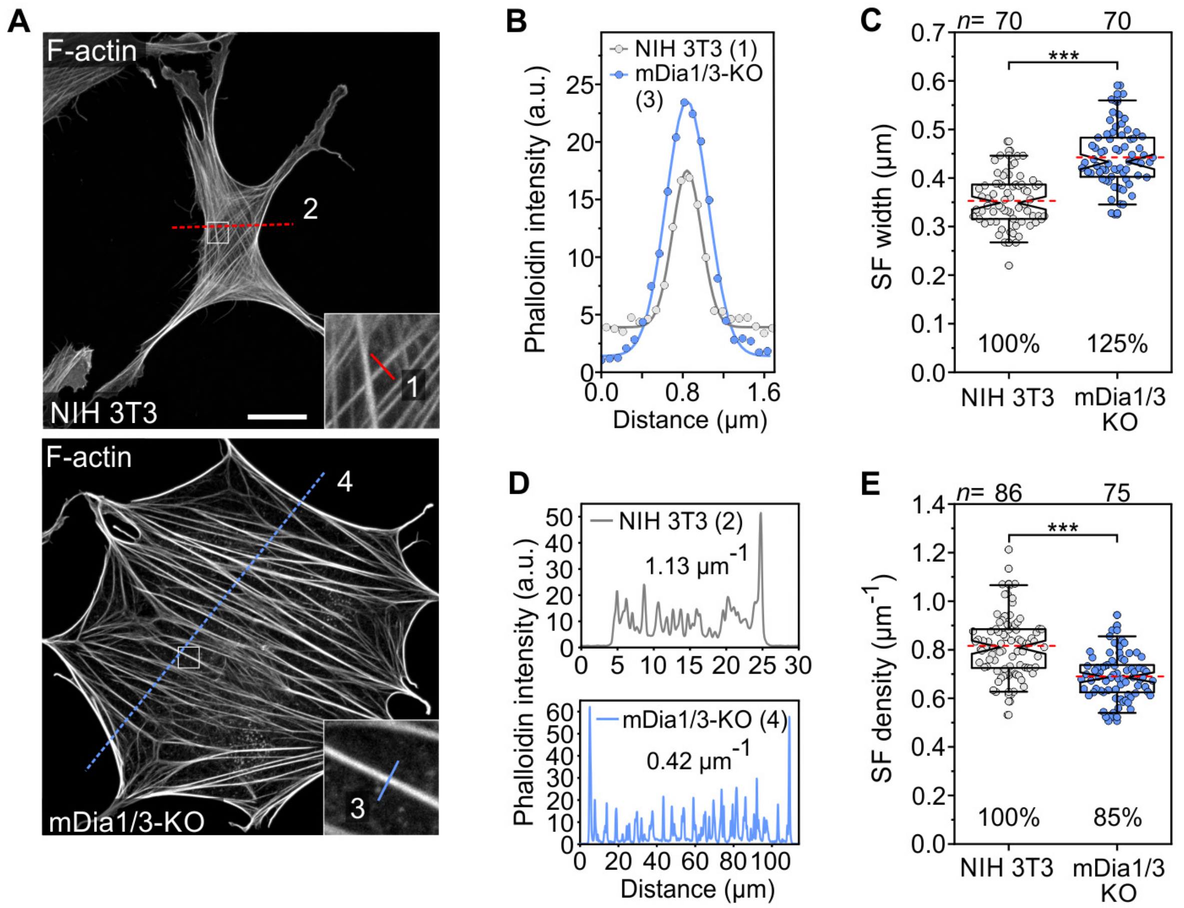

Fig. S9. Combined loss of mDia1/3 results in formation of thicker SFs. (**A**) Representative confocal z-stack projections of a control and an mDia1/3-KO cell seeded on FN and stained with phalloidin for the F-actin cytoskeleton. Insets, enlarged images of boxed regions. Scale bar, 20 µm. Dashed and solid lines indicate areas along which intensity profiles were created. (**B**) Quantification of phalloidin intensity across the two SFs in control and mutant cells shown in (A). Solid lines depict Gaussian fit of data points. (**C**) Quantification of SF width. (**D**) Phalloidin intensity profiles along dashed lines indicated in (A). Numbers in blots indicate mean SF number per micron. (**E**) Quantification of SF density in wild-type and mutant cells. (C,E) Boxes in box plots indicate 50% (25-75%) and whiskers 90% (5-95%) of all measurements, with dashed red lines depicting the means. Medians are highlighted by indentation of boxes. Student's t-test. Results are pooled data from 3 biologically independent replicates. *n*, number of cells analyzed. Percentages are shown to illustrate the differences between cell lines. ***$P < 0.001$.

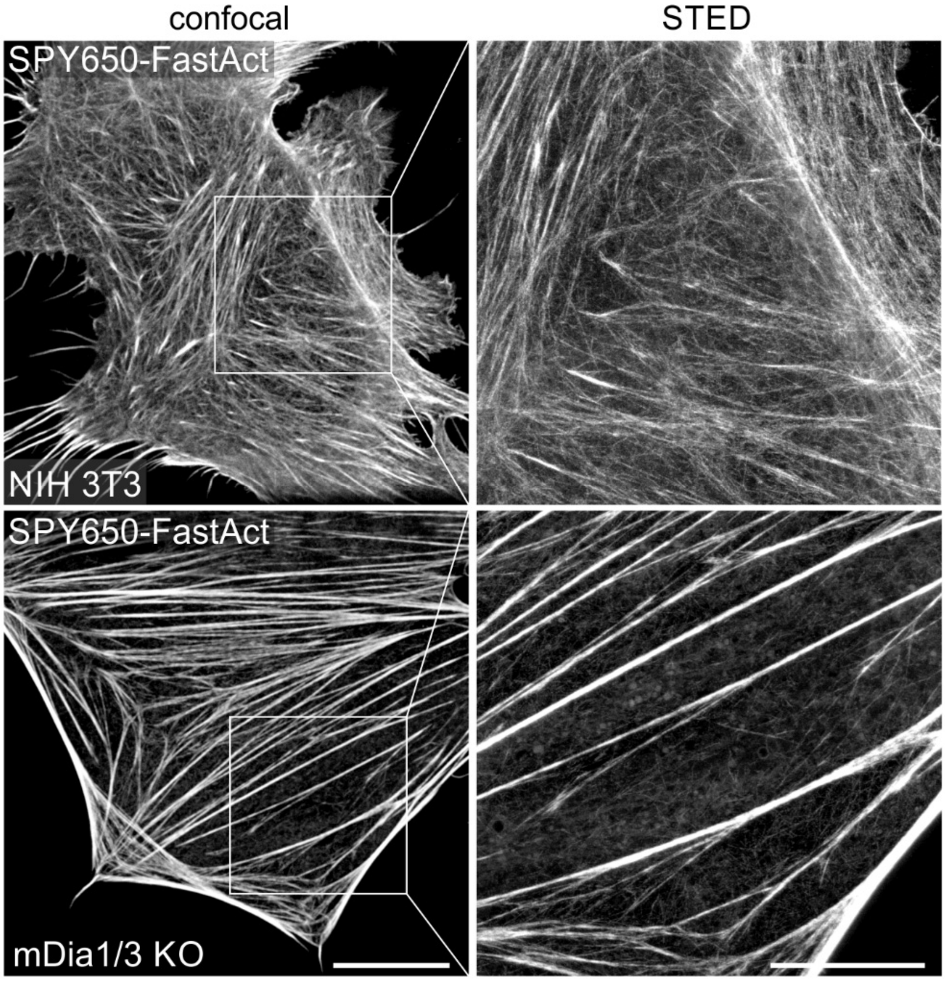

Fig. S10. mDia1/3-deficient fibroblasts exhibit dramatic changes in F-actin cytoskeleton architecture. Representative confocal images of live control and mDia1/3-KO cells on FN stained with the live actin probe SPY650-FastAct are shown left. Enlarged live STED images of the boxed regions marked on the left are shown on the right. These experiments correspond to data with fixed cells shown in Fig. 5A and B. Scale bar, 20 µm and inset, 10 µm.

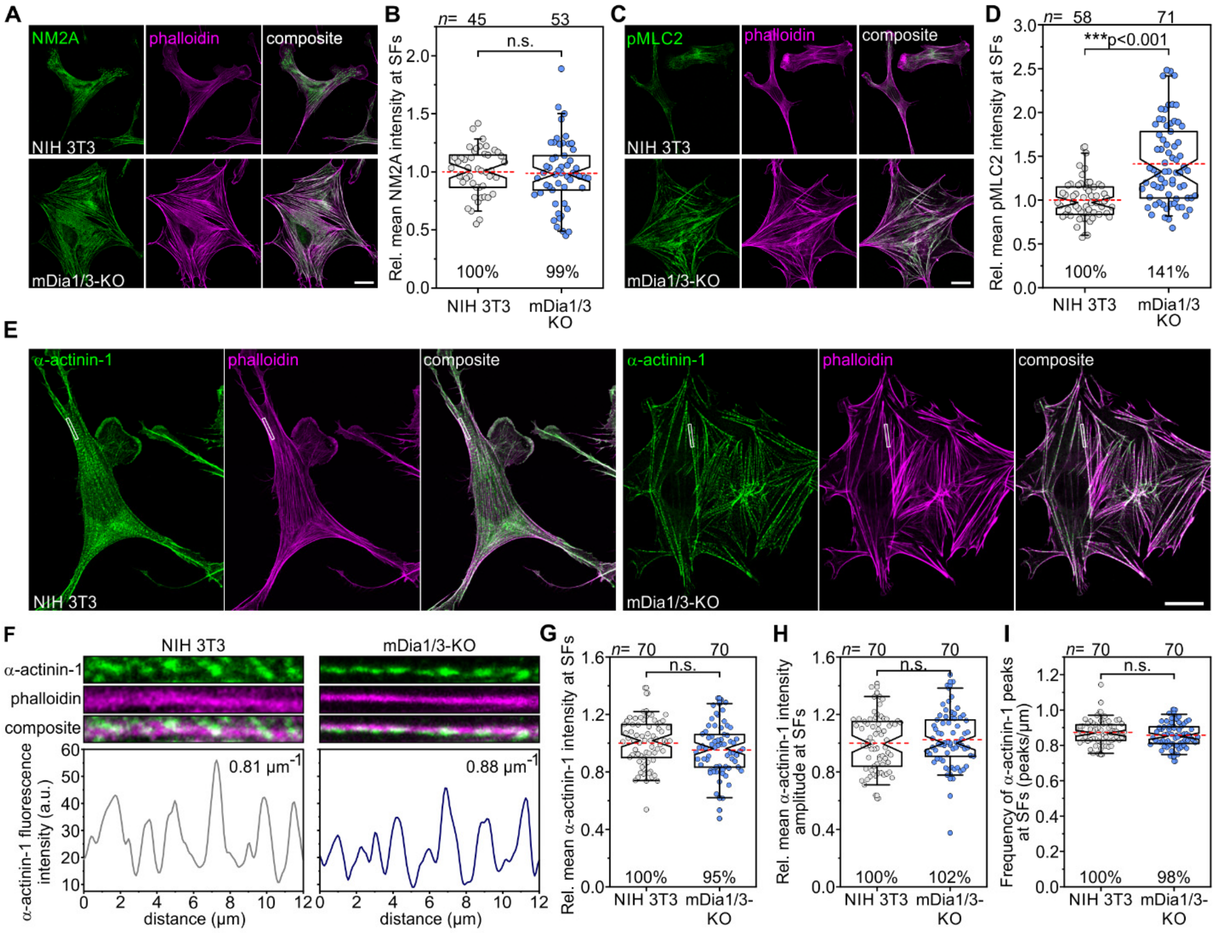

Fig. S11. mDia1/3-deficient fibroblasts exhibit increased SF contractility. (**A**) Representative images of a fixed control and an mDia1/3-KO cell on FN co-stained for the F-actin cytoskeleton and NM2A. Scale bar, 20 µm. (**B**) Quantification of NM2A intensity at SFs in control and mutant cells. (**C**) Representative images of a fixed control and an mDia1/3-KO cell on FN co-stained for the F-actin cytoskeleton and pMLC2. Scale bar, 20 µm. (**D**) Quantification of pMLC2 intensity at SFs in control and mutant cells. (**E**) Representative images of a fixed control and an mDia1/3-KO cell on FN co-stained for the F-actin cytoskeleton and α-actinin-1. Scale bar, 20 µm. (**F**) Upper panel: Enlarged images of white boxed regions shown in (F) depicting representative SFs. Lower panel: α-actinin-1 fluorescence intensity profiles from images shown above. Numbers indicate the frequency of α-actinin-1 peaks at SFs. (**G**) Quantification of the relative mean α-actinin-1 intensity at SFs. (**H**) Quantification of the relative mean amplitude of α-actinin-1 intensity peaks at SFs. (**I**) Quantification of the frequency of α-actinin-1 peaks along SFs. (C,D,G,H,I) Boxes in box plots indicate 50% (25-75%) and whiskers 90% (5-95%) of all measurements, with dashed red lines depicting the means. Medians are highlighted by indentation of boxes. Mean values and percentages are shown to better illustrate the differences between cell lines. *n*, number of cells analyzed. Mann-Whitney-U-test test. Results are pooled data from 3 biologically independent replicates. ****P* < 0.001. n.s., not significant.

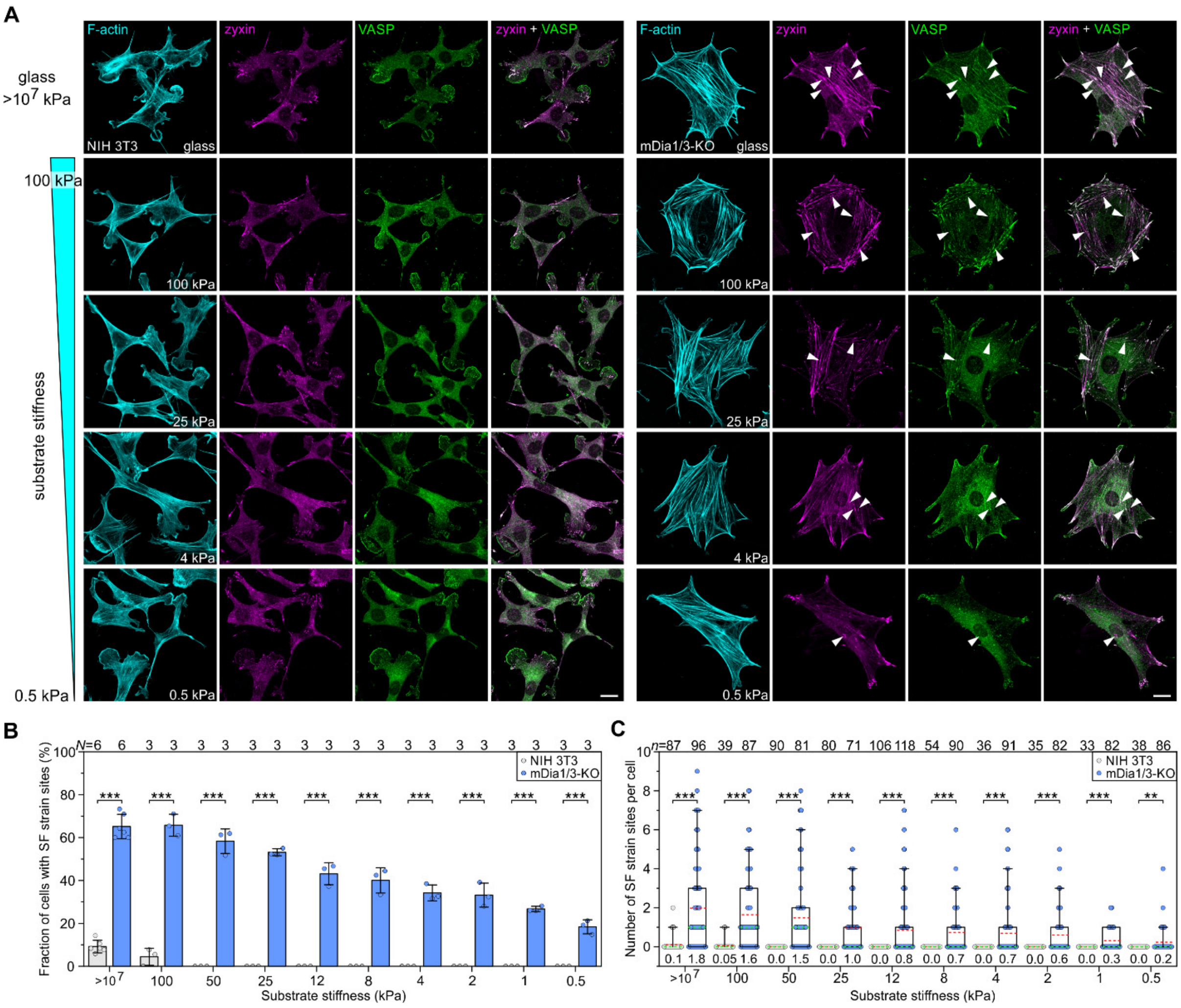

Fig. S12. SF strain site formation depends on substrate stiffness. (**A**) Representative images of a fixed control and mDia1/3-KO cells on FN-coated substrates of varying stiffness as indicated and co-stained for the F-actin cytoskeleton (cyan), zyxin (magenta) and VASP (green). Arrowheads indicate SF-strain sites. Scale bar, 20 µm. (**B**) Quantification of the proportion of cells forming strain sites. Bars and error bars represent means ± SD. Data points indicate individual experiments. Student's t-test. *N*, number of biologically independent replicates. (**C**) Quantification of strain sites per cell. Boxes in box plots indicate 50% (25-75%) and whiskers 90% (5-95%) of all measurements, with dashed red lines depicting the means. Medians are highlighted by dashed green lines. Mean values are shown to better illustrate the differences between cell lines. Mann-Whitney-U-test test. *n*, number of cells analyzed. (B,C) Results are pooled data from at least 3 biologically independent replicates. $^{**}P < 0.01$ and $^{***}P < 0.001$.

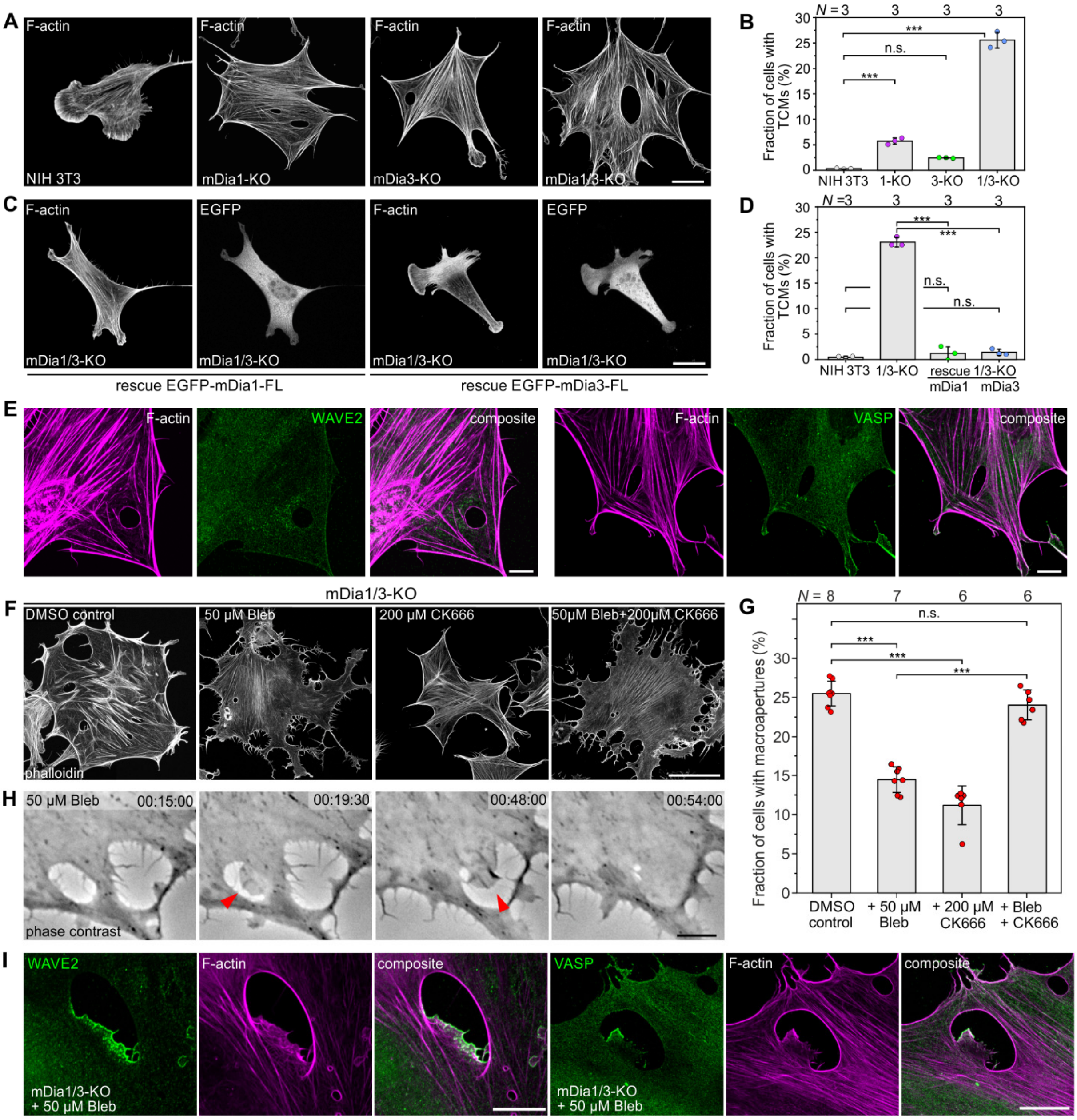

Fig. S13. Actomyosin contractility inhibits TCM closure by lamellipodial protrusion. (**A**) Confocal z-stack projections of phalloidin-stained control and mDia mutant cells, the latter of which form TCMs. Scale bar, 20 µm. (**B**) Quantification of TCM formation of cells shown in (A). (**C**) Expression of EGFP-tagged full-length (FL) mDia1 and mDia3 suppresses the formation of TCMs. Representative confocal z-stack projections of phalloidin-stained cells are shown. Scale bar, 20 µm. (**D**) Quantification of TCM formation in control, mDia1/3-KO and mDia1/3-KO cells expressing EGFP-tagged mDia1-FL or mDia3-FL. (**E**) Representative confocal z-stack projections of TCMs stained with phalloidin and co-stained for WAVE2 or VASP. Scale bars, 10 µm. (**F**) Representative confocal z-stack projections of phalloidin-stained mDia1/3-KO cells treated with the myosin 2 inhibitor blebbistatin (Bleb, 50 µM), the Arp2/3

complex inhibitor CK666 (200 µM) or both. DMSO-treated mDia1/3-KO cells are shown as control. (**G**) Quantification of macroaperture formation in control and treated cells displayed in (F). (**H**) Still images from a time-lapse, phase-contrast movie of a blebbistatin-treated mDia1/3-deficient cell on FN illustrating the closure of macroapertures by lamellipodia-like membrane extension (red arrow heads) into the void space; data correspond to movie S7. Time is indicated in h, min and sec. Scale bar, 10 µm. (**I**) Representative confocal z-stack projections of two macroapertures in blebbistatin-treated mDia1/3-KO cells stained with phalloidin and co-stained for WAVE2 or VASP. Scale bars, 25 µm. (B,D,G) Data represent mean ± SD. One-way ANOVA with Tukey Multiple Comparison test. Data points indicate means of individual experiments with at least 10 images each. *N*, number of biological replicates. ***$P < 0.001$. n.s., not significant.

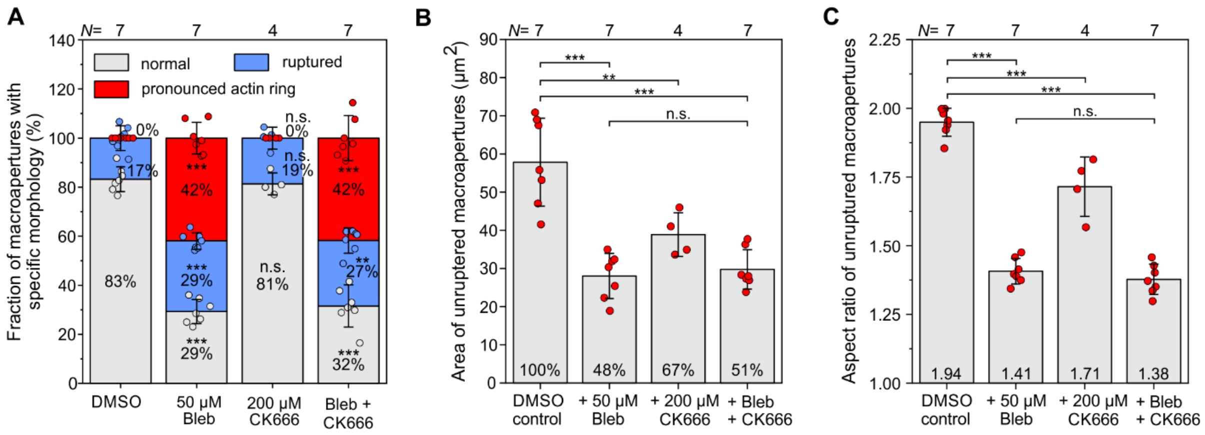

Fig. S14. Morphology of cellular macroappertures in mDia1/3-KO fibroblasts upon treatment with pharmalogical inhibitors. (**A**) Quantification of macroaperture morphology in the absence or presence of 50 µM blebbistatin (Bleb), 200 µM CK666 or both for 30 min. Data relate to fig. S10F. (**B**) Quantification of the size of unruptured macroapertures. (**C**) Quantification of the aspect ratio of unruptured macroappertures. (A-C) Bars and error bars represent means ± SD. Data points indicate means of individual experiments with at least 10 images each. *N*, number of biological replicates. One-way ANOVA with Tukey Multiple Comparison test. Percentages and mean values are shown to better illustrate the differences between cell lines. ***$P < 0.001$, **$P < 0.01$. n.s., not significant.

Table S1. List of DNA oligos used in the study.

**Primer Name**	**Sequence 5' → 3'**	**Purpose**
α-actinin-1_pEGFP-C1_Fw	CACCTCGAGCTATGGACCATTATGATTCCCAGCAG	mammalian expression
α-actinin-1_pEGFP-C1_Rev	CACGGTACCTTAGAGGTCGCTCTCGCCATACAG	mammalian expression
RhoA_CAAX_pEGFP-C1_Fw	GATCTTGCCTCATCTTGTGAG	mammalian expression
RhoA_CAAX_pEGFP-C1_Rev	GTACCTCACAAGATGAGGCAA	mammalian expression
α-actinin-1_pGEX-6P-3_Fw	CACGTCGACATGGACCATTATGATTCCCAGCAGAC	bacterial expression
α-actinin-1_pGEX-6P-3_Rev	CACGCGGCCGCTTAGAGGTCGCTCTCGCCATACAG	bacterial expression
FHL3_pGEX-6P-1_Fw	GCGGAATTCATGAGCGAGGCATTTGACTGT	bacterial expression
FHL3_pGEX-6P-1_Rev	CGCGTCGACTCAGGGGCCTGCTTGGCTGCA	bacterial expression
mDia1_GT_Fw	TGCAAGAAGGTAAAAGATTGGCT	genotyping
mDia1_GT_Rev	CCGACAGACAAATGCCCATA	genotyping
mDia3_GT_Fw	TGATTGCAGCTGCTTAGGG	genotyping
mDia3_GT_Rev	CCTTAGCTATGAGTGGGCTCC	genotyping

Table S2
Source data for experiments with small sample size.

Movie S1

Aspiration of control and mDia-KO mutant cells reveals markedly diminished cortical rigidity of the mDia1/3-double mutant. Time lapse, phase-contrast imaging of control NIH 3T3 and mutant cells at a stable suction pressure of 50 Pa shows that the initial indentation length (Lp) of mDia1/3-KO cells is considerably larger than of control and the single mutants. For more details, see Fig. 2A and B. Time is indicated in min and sec. Scale bar, 10 µm.

Movie S2

Combined loss of mDia1 and mDia3 results in extensive blebbing during mitosis. Phase-contrast imaging of dividing control and mDia1/3-KO cells on FN revealed excessive blebbing in the double mutant as compared to the NIH 3T3 control. For more details, see fig. S3A-C. Time is indicated in h, min and sec. Scale bar, 10 µm.

Movie S3

Development of the stretched morphology of mDia1/3-KO cells over time. This time lapse, phase-contrast movie of mDia1/3-KO cells shows the formation of the stretched phenotype over time following initial spreading on FN. For more details, see fig. S6F. Time is indicated in h, min and sec. Scale bar, 50 µm.

Movie S4

Combined loss of mDia1 and mDia3 in NIH 3T3 fibroblasts results in diminished FA turnover and increased FA sliding. Representative TIRF movies of an NIH 3T3 and mDia1/3-KO cell migrating on FN and displaying EGFP-paxillin accumulation in FAs. Note the relatively rapid turnover of FAs in NIH 3T3 wild-type cells as compared to the double mutant, in which the FAs are more stable and preferentially slide along the ECM. For more details, see Fig. 4. Time is indicated in h and min. Scale bar, 10 µm.

Movie S5

Combined loss of mDia1 and mDia3 in NIH 3T3 fibroblasts induces the formation of transcellular macroapertures. This time lapse, phase-contrast movie of a migrating mDia1/3-KO double-mutant cell on FN shows the formation and closure of a transcellular macroaperture. For more details, see Fig. 7A. Time is indicated in h, min and sec. Scale bar, 50 µm.

Movie S6

Dynamics of TCM closure. This time-lapse, TIRF movie of an mDia1/3-KO cell expressing EGFP-tagged α-actinin-1 shows that constriction of the sarcomere-like subunits at the TCM periphery coincides with closure, strongly suggesting that this process is driven by actomyosin-based contraction. For more details, see Fig. 7I. Time is indicated in h and min. Scale bar, 5 µm.

Movie S7

Conversion of TCMs into TEM-like structures. This time-lapse, phase contrast movie of an mDia1/3-KO cell forming TCM shows that treatment of the mutant cells with 50 µM blebbistatin (Bleb) converts the elliptic macroapertures into circular TEM-like structures that invade the void space by lamellipodia-like structures. For more details, see fig. S10H. Time is indicated in h, min and sec. Scale bar, 25 µm.

Movie S8
Extensive pulling forces precede the formation of TCMs. This time-lapse, phase-contrast movie of an mDia1/3-KO double mutant on FN displays extensive pulling forces acting of the cytoskeleton and the membrane prior to the formation of a TCM. For more details, see Fig. 8A. Time is indicated in h, min and sec. Scale bar, 20 μm.

Movie S9
Rupture of SFs typically precedes TCM formation. This time-lapse, TIRF movie of an mDia1/3-KO double-mutant expressing mScarlet-LifeAct and the membrane marker EGFP-CAAX on FN shows strain-mediated rupture of prominent SF bundles followed by expansion of this area leading to the formation of a TCM. Note that the rupture of the plasma membrane occurs about 1 min after the SF rupture. For more details, see Fig. 8B. Time is indicated in min and sec. Scale bar, 10 μm.

Movie S10
Formation of a large TCM globally releases tension to boost protrusion of lamellipodia. This time-lapse, phase-contrast movie of an mDia1/3-KO double mutant on FN shows the formation of a large TCM, which is immediately followed by a burst of protruding lamellipodia due to the reduced tension of the membrane. Note that the formation of the TCM occurs in a very thin region of the mutant cell, as evidenced by the absence of the thread-like mitochondria, where the ventral and dorsal parts of the plasma membrane come into close proximity to each other, facilitating membrane fusion. For more details, see Fig. 8C. Time is indicated in h, min and sec. Scale bar, 10 μm.

Movie S11
Hypotonic shock promotes TCM formation exclusively in mDia1/3-KO cells. Representative time-lapse, phase-contrast movies of an NIH 3T3 control and an mDia1/3-KO cell prior and after hypotonic shock. Note, induction of TCM formation occurred immediately after hypotonic shock in the double mutant, but not in the control cell. For more details, see Fig. 8G und H. Time is indicated in min and sec, Scale bar, 25 μm.
